# The biology of SCUBE

**DOI:** 10.1186/s12929-023-00925-3

**Published:** 2023-05-26

**Authors:** Yuh-Charn Lin, Binay K. Sahoo, Shiang-Shin Gau, Ruey-Bing Yang

**Affiliations:** 1 Department of Physiology, School of Medicine, College of Medicine, Taipei Medical University, Taipei, Taiwan; 2 Institute of Biomedical Sciences, Academia Sinica, Taipei, Taiwan; 3 Biomedical Translation Research Center, Academia Sinica, Taipei, Taiwan; 4 Program in Drug Discovery and Development Industry, College of Pharmacy, Taipei Medical University, Taipei, Taiwan

**Keywords:** Biomarker, Coreceptor, Endothelial cells, Signal transduction, SCUBE

## Abstract

The SCUBE [Signal peptide-Complement C1r/C1s, Uegf, Bmp1 (CUB)-Epithelial growth factor domain-containing protein] family consists of three proteins in vertebrates, SCUBE1, 2 and 3, which are highly conserved in zebrafish, mice and humans. Each *SCUBE* gene encodes a polypeptide of approximately 1000 amino acids that is organized into five modular domains: (1) an N-terminal signal peptide sequence, (2) nine tandem epidermal growth factor (EGF)-like repeats, (3) a large spacer region, (4) three cysteine-rich (CR) motifs, and (5) a CUB domain at the C-terminus. Murine *Scube* genes are expressed individually or in combination during the development of various tissues, including those in the central nervous system and the axial skeleton. The cDNAs of human SCUBE orthologs were originally cloned from vascular endothelial cells, but SCUBE expression has also been found in platelets, mammary ductal epithelium and osteoblasts. Both soluble and membrane-associated SCUBEs have been shown to play important roles in physiology and pathology. For instance, upregulation of SCUBEs has been reported in acute myeloid leukemia, breast cancer and lung cancer. In addition, soluble SCUBE1 is released from activated platelets and can be used as a clinical biomarker for acute coronary syndrome and ischemic stroke. Soluble SCUBE2 enhances distal signaling by facilitating the secretion of dual-lipidated hedgehog from nearby ligand-producing cells in a paracrine manner. Interestingly, the spacer regions and CR motifs can increase or enable SCUBE binding to cell surfaces via electrostatic or glycan-lectin interactions. As such, membrane-associated SCUBEs can function as coreceptors that enhance the signaling activity of various serine/threonine kinase or tyrosine kinase receptors. For example, membrane-associated SCUBE3 functions as a coreceptor that promotes signaling in bone morphogenesis. In humans, SCUBE3 mutations are linked to abnormalities in growth and differentiation of both bones and teeth. In addition to studies on human SCUBE function, experimental results from genetically modified mouse models have yielded important insights in the field of systems biology. In this review, we highlight novel molecular discoveries and critical directions for future research on SCUBE proteins in the context of cancer, skeletal disease and cardiovascular disease.

## Introduction

Three SCUBE [Signal peptide-complement C1r/C1s, Uegf, and Bmp1 (CUB)-Epithelial growth factor domain-containing proteins] proteins in vertebrates comprise a small family with critical functions in a wide variety of physiological and pathological processes. The human *SCUBE* genes were originally identified from cultured endothelial cells (ECs), but rodent homologs were identified on the basis of their distinctive embryonic expression patterns. Over the past two decades, significant progress has been made in understanding the functions of SCUBEs in normal physiology and disease. In this review, we provide a detailed survey of the crucial known and emerging roles of SCUBE proteins. We begin by recounting the identification, phylogenetic relationships, and protein domain organization and functions of the proteins. Then for each SCUBE family member, we highlight the gene expression patterns, posttranslational control, developmental and physiological functions, and associated diseases. We also discuss several potential translational and therapeutic applications as well as future research directions related to the SCUBE family.

## Discovery and phylogenetic analysis

**Fig. 1:**
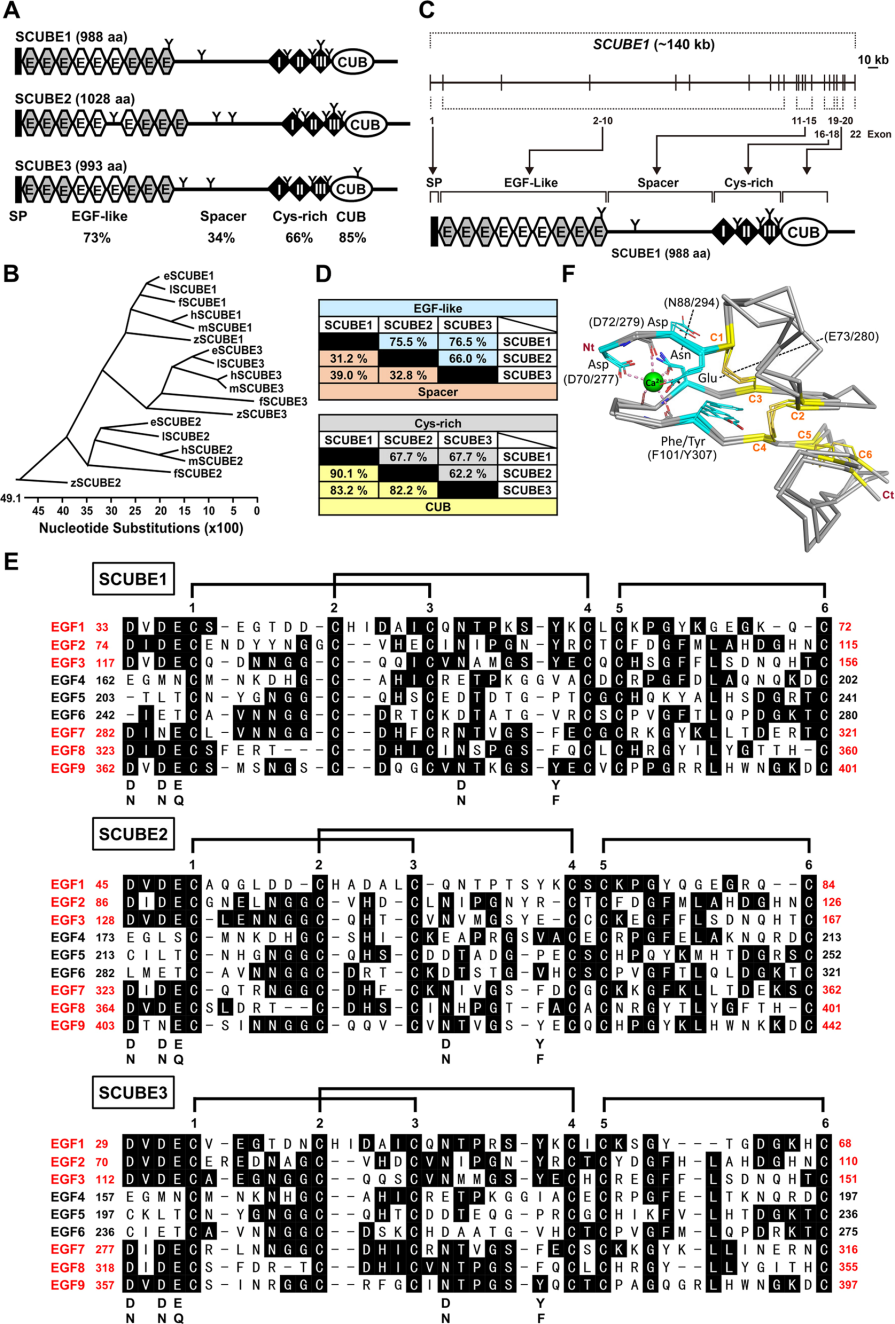
Protein domains, phylogenetic analysis, genomic organization, sequence alignment, and sequence identity of SCUBE protein family. **A** Graphic illustration of the domain structure of human SCUBE1, 2 and 3 (see Table 1). SP, signal peptide sequence; E, EGF-like domain (grey shade indicated the calcium-binding EGF module); Cys-rich, cysteine-rich repeats; CUB, the CUB domain. “Y” marks the potential N-linked glycosylated sites of each SCUBE protein. Protein domain sequence identity shared among human SCUBE members is calculated at the bottom; the highest homology was found in the CUB domain (83%), followed by the EGF-like (73%) and Cys-rich (66%) domains. The spacer region appears to have the lowest homology (34%) and may be associated with the unique functions of each SCUBE member. **B** Phylogenetic tree of the SCUBE family. Similarity of human (h), mouse (m), zebrafish (z), eagle (e), lizard (l), or frog (f) SCUBE protein sequences was analyzed by using the Lasegene MEGALIGN program (ClustalW algorithm). The length of each pair of branches represents the phylogenic distance between sequence pairs. Below the tree is a scale indicating the number of “Nucleotide Substitutions” for both DNA and protein sequences. **C** Genomic structure of human *SCUBE1*. *SCUBE1* gene consists of 22 exons spanning about 140 kb on chromosome 22. The exon–intron boundaries are well preserved among the SCUBE gene members, which suggests possible gene duplication during evolution. Of note, *SCUBE* genomic organizations derived from zebrafish, mouse and human genomes (http://genome.ucsc.edu) follow a modular arrangement, with the signal peptide sequence, each epidermal growth factor (EGF)-like domain, and Cys-rich motif, each encoded by a single exon. In addition, the spacer region is encoded by five exons and the CUB is encoded by two exons. **D** Amino acid sequence identity of SCUBE family. Data in the upper right (blue shading) represent the sequence identity in EGF-like domain, and data on the bottom left (orange shading) represent the sequence identity in spacer region between SCUBE proteins (upper panel). Data on the upper right (gray shading) present the sequence identity in Cys-rich domain, and data on the bottom left (yellow shading) present the sequence identity in CUB domain between SCUBE proteins (lower panel). **E** Sequence alignment, sequence identity, and structural comparison of EGF-like repeats of human SCUBE proteins. Protein sequences of SCUBE1, SCUBE2 and SCUBE3 used for alignment were derived from NCBI Reference Sequence Database, as listed in Table 1. Symbols use the one-letter code for amino acids. A dash indicates a gap. The conserved cysteines are numbered 1 to 6, and the predicted disulfide bonds form as follows: C1-C3, C2-C4, and C5-C6. Amino acids indicated below highlight the canonical calcium binding (cb) consensus sequence (D/N)-X-(D/N)-E/Q-X_*m*_ (D/N^*^) X_*n*_ (Y/F), where *m* and *n* are variable and the asterisk indicates potential β-hydroxylation [17]. Of note, two triplets of cbEGF_1-3_ or cbEGF_7-9_ modules (amino acid residue boundaries marked in red fonts) are well conserved in all SCUBE members. **F** Structural comparison of SCUBE3 second and seventh cbEGF-like domains with human NOTCH1 fifth cbEGF-like domain. 3D models of the second and seventh EGF-like domains of human SCUBE3 obtained from AlphaFold2 (AF-Q8IX30-F1-model_v3) [187] were superimposed on the fifth cbEGF-like domain of human NOTCH1 (PDB 5FM9) [221]. These EGF-like domains contain a short calcium binding motif sequence signature: (D/N)-X-(D/N)-E/Q-X_*m*_-(D/N^*^)-X_*n*_-(Y/F), where *m* and *n* are variable and the asterisk indicates potential β-hydroxylation [17]. This canonical calcium coordination module is found near at the N-terminus. The residues coordinating calcium ion (in green) are shown as sticks, and the coordination as pink dotted lines. The five calcium-binding motif residues indicated in **E** are presented as cyan sticks; Asn and Phe/Try between C3 and C4 of these EGF-like domains are consistent with the substrate sequence pattern of Asp/Asn-β-hydroxylase [222, 223]. The three paired cysteines (C1:C3, C2:C4, and C5:C6) are presented as yellow sticks. Atoms are colored, with oxygen in red, nitrogen in blue and sulfur in gold. This figure was produced using PyMOL (Schrödinger, LLC. The PyMOL molecular graphics system, version 1.8, https://pymol.org). Nt, NH_2_ terminus; Ct, COOH terminus

The first identified SCUBE family member (SCUBE1) was isolated from developing mouse urogenital ridge [1] and human vascular endothelial [2] cDNA libraries. Subsequent genomic and homology searches led to the discovery of two additional family members, SCUBE2 [3, 4] and SCUBE3 [5] (Fig. 1A). Based on a comprehensive search using the Genome Browser website (http://genome.ucsc.edu), the three *SCUBE* genes were found to be highly conserved in zebrafish, mice and humans. Thus, the three proteins comprise a small, evolutionarily conserved family (Fig. 1B) (Table 1) [1–10]. Despite their conservation in vertebrates, homologous genes have not been identified in the genomes of single-cell yeast (*Saccharomyces cerevisiae*) or invertebrate animals, such as fruit flies (*Drosophila melanogaster*) or nematodes (*Caenorhabditis elegans*). This species distribution is in line with the idea that *SCUBE* genes might be involved in the development or function of anatomical features specific to vertebrates or animals with vasculature, such as the neural tube or blood vessels. Moreover, comparative genetic analyses revealed that the organization of *SCUBE* genes in zebrafish, mouse and humans consistently follows a modular arrangement. The N-terminal signal peptide, every epidermal growth factor (EGF)-like domain, and single cysteine-rich (CR) motifs are each encoded by single exon. Meanwhile, the spacer region is encoded by five exons, and the CUB domain is encoded by two exons (Fig. 1A, C).

**Table 1:** Reference sequences for human, mouse and zebrafish SCUBE family

Species	Gene	Chr. location	cDNA	Protein	AA	References
Human	*SCUBE1*	22q13.2	NM_173050	NP_766638	988	[2]
Human	*SCUBE2*	11p15.4	NM_001367977	NP_001354906	1028	[4]
Human	*SCUBE3*	6p21.2	NM_152753	NP_689966	993	[5]
Mouse	*Scube1*	15qE1	NM_022723	NP_073560	988	
Mouse	*Scube2*	7qE3	NM_020052	UniProt Q9JJS0 + NP_064436	1025^a^	
Mouse	*Scube3*	17qA3.3	NM_001004366	NP_001004366	993	
Zebrafish	*scube1*	Chr. 4	NM_001328599	NP_001315528	1024	[8]
Zebrafish	*scube2*	Chr. 7	NM_001014813	NP_001014813	1010	[6, 7, 10]
Zebrafish	*scube3*	Chr. 23	NM_001260501	NP_001247430	995	[9]

*Chr* chromosome; *AA* amino acids; *Ref* references

^a^Full-length coding sequence for mouse *Scube2* was derived by merging two spliced transcripts

The *SCUBE* genes give rise to polypeptides of about 1000 amino acids with five major protein domains: (1) an N-terminal leader sequence, (2) nine tandem EGF-like repeats, (3) a large spacer region that undergoes N-glycosylation, (4) three stretches of six cysteine residues (designated CR motifs) with conserved amino acid spacing between the cysteines, and (5) a CUB domain at the C-terminus (Fig. 1A). The amino acid sequence of SCUBE3 is 65.6% identical with SCUBE1 and 58.5% identical with SCUBE2, while SCUBE1 and SCUBE2 share 65.6% identity. The CUB domain is the most highly conserved region, sharing 82.2–90.1% (~ 85.2%) identity among all three members. The EGF-like repeats range from 66.0 to 76.7% (~ 72.7%) identity and CR motif region is 62.2–67.7% (~ 65.8%) identical among all three members (Fig. 1D). The spacer region is the most diverse (34% identity), which suggests that this region might be centrally involved in molecular functions that are uniquely associated with individual SCUBE members. In the next section, we provide further details about the overall structures and functions of each SCUBE protein domain.

## Domain organization and function

### Signal peptide and EGF-like domain

SCUBE proteins have an N-terminal signal peptide domain that is important for protein secretion and localization to the membrane. The adjacent EGF-like domains are composed of stretches of about 30–40 residues and are located near the N-terminus of the SCUBE protein. The EGF-like domain is an evolutionarily conserved protein domain that was first identified in EGF but also exists in many other secreted or membrane-localized proteins. These EGF-like domain-containing proteins are mostly of animal origin and include growth factors, adhesive selectin molecules, extracellular matrix protein, and clotting factors [11]. The domain is frequently found as tandem copies with various degrees of conservation. These tandem repeats tend to fold together to become a single linear solenoid structure that works as a functional unit. In general, each EGF-like domain contains six cysteine residues that form three intramolecular disulfide bonds in a conserved pattern (i.e., C1–C3, C2–C4 and C5–C6) [11] (Fig. 1E). Whether the EGF-like domains are present in the extracellular portion of a membrane protein or in a secreted protein, a typical function of the domain is to facilitate homophilic [12, 13] or heterophilic protein–protein interactions, such as thrombin–thrombomodulin [14], Notch1-Jagged-1 [15], and low-density lipoprotein receptor–proprotein convertase subtilisin/kexin type 9 [16] interactions. In addition, some specialized EGF-like domains are capable of binding calcium and are thus termed calcium-binding EGF (cbEGF) domains. These cbEGF domains carry the characteristic six cysteine residues but also have an additional calcium-binding motif. The motif sequence is: (D/N)–X–(D/N)–E/Q–X_*m*_–(D/N*)–X_*n*_-(Y/F), where *m* and *n* are variable and the asterisk indicates a potential β-hydroxylation site [17] (Fig. 1F). Extracellular cbEGFs are known to participate in protein–protein and protein–carbohydrate interactions [18]. A previous biochemical study showed that triple cbEGF repeats could form a rigid, protease-resistant conformation upon calcium binding [19]. Therefore, when a cbEGF domain-containing protein is secreted or exposed to the calcium-rich extracellular environment, tandem cbEGF repeats may acquire a stiff, protease-resistant structure that can facilitate protein–protein interactions.

Intriguingly, we found that six of the nine EGF-like repeats in SCUBE proteins contain calcium-binding consensus sequences and are organized in triplet modules (i.e., cbEGF_1-3_ or cbEGF_7-9_) (Fig. 1A, E). When overexpressed, these SCUBE proteins are secreted glycoproteins that can form homomeric or heteromeric complexes with other SCUBE proteins via EGF-like domain-mediated interactions [2, 5, 20, 21] (Table 2) and are stably associated with the cell surface [2, 4, 5]. Further deletion mapping and cell aggregation assays revealed that the EGF modules are involved in reciprocal (*trans*) or lateral (*cis*) interactions between SCUBE molecules [20, 22]. Moreover, the triplet cbEGF_7–9_ comprises a minimal unit (i.e., sufficient but not necessary) for the calcium-dependent *trans*-homophilic association of SCUBE1 with adjacent cells [20]. In addition, the EGF-like repeats in SCUBE proteins can interact with and modulate signaling activities of growth factors. For instance, SCUBE2 interacts with vascular endothelial growth factor (VEGF) [23, 24], and SCUBE3 interacts with fibroblast growth factor 8 (FGF8) (Table 2) [9].

**Table 2:** Partners interacting with SCUBE members in biological and pathological processes

SCUBE proteins	Interacting proteins	Interacting domain	Functions	Molecular events	References
SCUBE1	SCUBE1	EGF-like	Platelet agglutination	Formation of homo-oligomeric complexes	[2, 20]
	SCUBE2			Formation of hetero-oligomeric complexes	[2]
	SCUBE3			Formation of hetero-oligomeric complexes	[5, 21]
	BMP2		Brain formation and zebrafish primitive hematopoiesis	When co-expressed in “signal-producing” cells, the C-terminal CR and CUB domains of SCUBE1 interact with BMP2 to function as a BMP antagonist. When expressed in “signal-receiving” cells, zebrafish Scube1 forms a complex with Bmp ligands and its receptors, behaving as a BMP coreceptor to augment BMP signaling activity	[8, 20]
	PEAR1	EGF-like and CUB		SCUBE1 binds PEAR1 through EGF-like repeats and CUB domain, possibly for platelet agglutination	[72]
	Caveolin-1	Spacer and CUB		SCUBE1 forms a complex with caveolin-1 via Spacer and CUB domains for its recruitment into lipid raft microdomains	[26]
	VEGFR2		Zebrafish angiogenesis	SCUBE1 cannot interact with VEGF, which suggests that SCUBE1 may not function as a coreceptor for VEGF	[23]
	BMP7		Protects against AKI	SCUBE1 acts as a BMP coreceptor by binding to BMP7 and its receptors and facilitating BMP7 signaling	[49]
	BMPRIB				
	BMPRII				
	FLT3 ligand	Spacer and CUB	Promotes AML	SCUBE1 might form a complex with FLT3 ligand and FLT3 via Spacer and CUB domains to facilitate pathological FLT3-LYN signaling	[44]
	FLT3	Spacer and CUB			
SCUBE2	SCUBE1			Formation of hetero-oligomeric complexes	[2]
	SCUBE2			Formation of homo-oligomeric complexes	[2]
	SCUBE3			Formation of hetero-oligomeric complexes	[21]
	BMP2	CR and CUB	Acts as breast tumor suppressor	The C-terminal region of SCUBE2 binds BMP protein and acts as a BMP antagonist	[112]
	SHH	CUB	Endochondral bone formation	SCUBE2 can specifically interact with SHH, IHH, and PTCH1 and enhance the HH signaling activity within the cholesterol-rich raft microdomains of the plasma membrane (HH signaling enhancer)	[4, 45]
	IHH	CUB			
	PTCH1	Spacer and CUB			
	Caveolin-1	CUB			
	E-cadherin	EGF-like	Acts as breast tumor suppressor	1. SCUBE2 co-localizes with E-cadherin at MCF-7 cell contacts, acting as a tumor suppressor for breast cancer 2. SCUBE2 forms a complex with E-cadherin mainly through its EGF-like repeats and inhibits breast-cancer cell migration and invasion via the reversal of EMT	[22, 51]
	VEGF	EGF-like	Promotes tumor angiogenesis	1. SCUBE2 colocalizes and interacts with VEGFR2 in HUVECs 2. SCUBE2 directly binds to VEGF-A_165_ and VEGFR2	[23]
	VEGFR2	CUB			
SCUBE3	SCUBE1			Formation of hetero-oligomeric complexes	[5, 21]
	SCUBE2			Formation of hetero-oligomeric complexes	[21]
	SCUBE3			Formation of homo-oligomeric complexes	[5, 21]
	TGF-β1		Promotes cardiac hypertrophy	SCUBE3 forms a complex with TGF-β1	[170]
	TβR-II	CUB	Promotes lung cancer progression	SCUBE3 binds to TβR-II via its C-terminal CUB domain, promoting lung cancer via EMT	[48]
	FGF8	EGF-like, Spacer, and CUB	Zebrafish fast muscle development	SCUBE3 interacts with FGF8/FGFR4 to enhance FGF8 signaling	[9]
	FGFR4	Spacer and CUB			
	BMP2	CUB	Craniofacial and skeletal development	SCUBE3 binds to BMP and BMP receptors, acting as an enhancer for BMP signaling; involved in BMP-dependent osteoblast differentiation	[21]
	BMP4				
	BMP7				
	BMPRIA				
	BMPRIB				
	BMPRII				
	VEGFR2			SCUBE3 cannot interact with VEGF, which suggests that SCUBE3 may not function as a coreceptor for VEGF	[23]

*AKI* acute kidney injury; *AML* acute myeloid leukemia; *BMP2* bone morphogenetic protein 2; *BMP4* bone morphogenetic protein 4; *BMP7* bone morphogenetic protein 7; *BMPRIA* BMP type IA receptor; *BMPRIB* BMP type IB receptor; *BMPRII* BMP type II receptor; *EMT* epithelial–mesenchymal transition; *FGF8* fibroblast growth factor 8; *FGFR4* fibroblast growth factor receptor 4; *FLT3* Fms-like receptor tyrosine kinase 3; *HUVECs* human umbilical vein endothelial cells; *IHH* Indian hedgehog; *PEAR1* platelet endothelial aggregation receptor-1; *PTCH1* patched homolog 1; *SCUBE* signal peptide-CUB-EGF domain-containing protein; *SHH* sonic hedgehog; *TβR-II* transforming growth factor-β type II receptor; *TGF-β1* transforming growth factor β-1; *VEGF* vascular endothelial growth factor; *VEGFR2* vascular endothelial growth factor receptor 2

### Spacer region

**Fig. 2:**
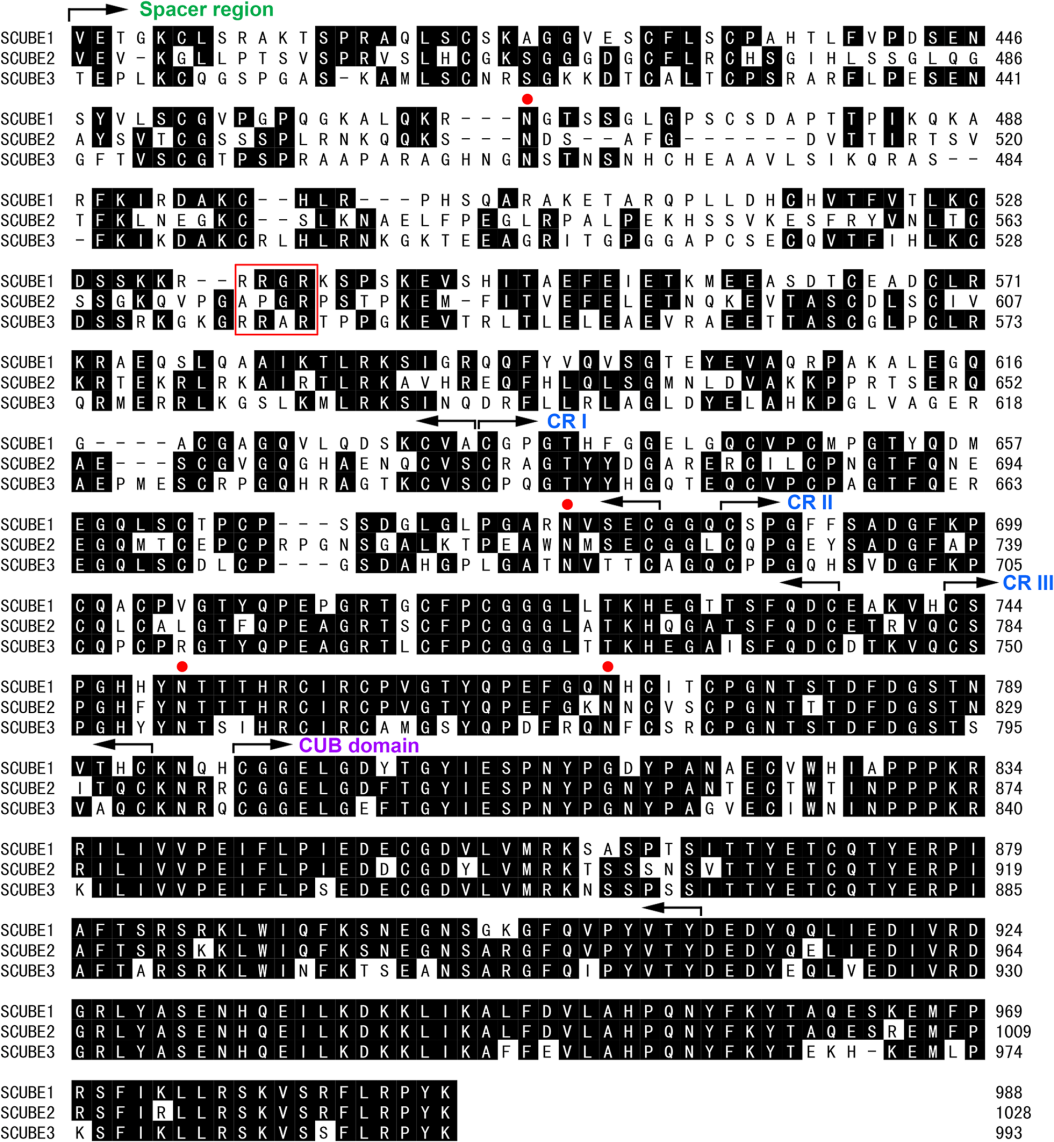
Sequence alignment of the spacer regions, Cys-rich domains, and CUB domains in the human SCUBE family. The margins of the spacer region and the CUB domain are derived from PFAM analysis and are marked by arrows. Amino acids residues that are identical in at least 2 protein sequences are highlighted in black. Potential N-linked glycosylation sites shared among the SCUBE proteins are denoted with a filled red circle. The minimal furin cleavage sites (RXXR), which are present in SCUBE1 and SCUBE3 but not SCUBE2, are boxed

The spacer region is a relatively large domain situated in the middle of the SCUBE protein, separating the EGF-like domains near the N-terminus from the CR repeats and CUB domain. The spacer is encoded by five exons and respectively contains 231, 227 and 241 amino acid residues in human SCUBE1, 2 and 3 proteins. A potential *N*-linked glycosylation motif (N–X–S/T consensus sequence, where X is any amino acid except proline) [25] is conserved within the spacer domains of all three SCUBE members (Figs. 1A and 2). To date, no detailed structural information has been published regarding the spacer region. However, our cellular and molecular analyses revealed that this domain functions to tether SCUBE1 protein to the plasma membrane, and it is also necessary for SCUBE1 secretion [20, 26]. Furthermore, we identified a stretch of positively charged residues within the spacer region (amino acids 501–550 of SCUBE1) that interacts electrostatically with anionic heparan sulfate proteoglycans on the plasma membrane [26]. Although it has not been confirmed experimentally, SCUBE2 or SCUBE3 might both utilize similar mechanisms for membrane anchorage, as stretches of positively charged residues were also found in the spacer regions of these molecules.

### Cysteine-rich domain

Between the spacer domain and the CUB domain lies the CR domain, which consists of three repeated motifs with six cysteine residues in each repeat. Of note, this 51-amino-acid motif with six cysteines bears little resemblance to the EGF-like domains (~ 40 amino acid residues). An important feature of the three CR motifs is that they can undergo *N*-linked glycosylation, as each motif in a SCUBE family member carries up to five potential well-conserved *N*-glycosites (Fig. 1A and Fig. 2). While the precise functions and structures of these motifs are currently unknown, our biochemical and molecular studies revealed that the CR domains are required for extracellular secretion and cell-surface expression of SCUBE1 in vitro [2, 20]. Most importantly, N-glycosylation at four different Asn residues (positions 679, 750, 779 and 789) within the CR domain appears to be essential for SCUBE1 cell-surface binding activity [26]. Further mutagenesis analysis verified that three of the four *N*-glycosites (i.e., Asn^679^, Asn^750^, and Asn^789^) are indeed utilized in vivo, and any given *N*-glycan is sufficient to allow the CR motif to bind the cell surface, possibly via an as yet unidentified glycan-lectin interaction [26].

### CUB domain

Following the CR domain, a SCUBE protein contains a single CUB domain and a highly conserved C-terminal tail (Figs. 1A, 2). The CUB domains are named after the proteins in which they were originally discovered: human complement proteases C1r and C1s, embryonic sea urchin protein Uegf, and human bone morphogenetic protein (BMP) 1 [27]. A 110-amino-acid module known as the CUB domain is nearly always present in extracellular and plasma membrane proteins. CUB domain-containing proteins participate in a wide variety of biological processes, including developmental patterning [28], axon guidance and angiogenesis [29], cell signaling [30], receptor-mediated endocytosis [31], hemostasis [32], fertilization [33], neurotransmission [34], tissue repair [35], tumor suppression [36], complement activation [37], and inflammation [38]. Although the functional roles of many CUB domains are largely unknown, their structures typically exhibit a β-sandwich fold [39] and some can mediate protein–protein interactions [40], homodimerization [37, 41], heterodimerization [33], and participate in the recognition of substrates and binding partners [42]. In a subset of CUB domains endowed with calcium-binding activity (i.e., cbCUBs), coordination of the calcium ion involves three acidic residues (one glutamic acid and two aspartic acid residues). Structural studies provided further evidence that calcium binding induces and stabilizes the conformation of the cbCUB domain, potentially facilitating its interaction with protein ligands [42, 43].

**Fig. 3:**
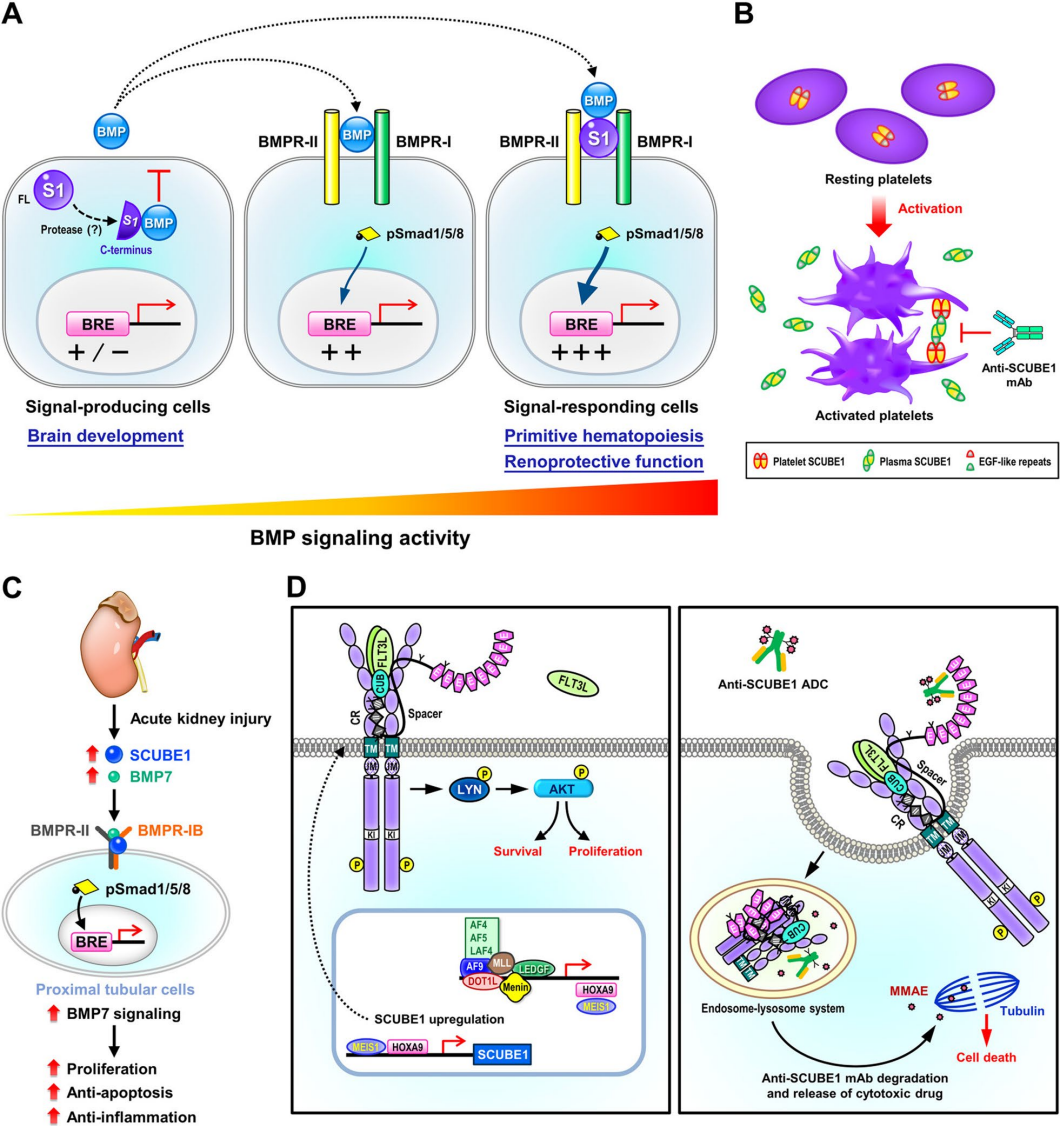
Pathological involvement of SCUBE1. **A** SCUBE1 modulates BMP signaling activity in a context-dependent manner. When expressed in “signal-producing” cells, the C-terminal CR and CUB domains are released by an as yet undetermined proteinase, allowing them to bind and inhibit the secretion of mature BMP into the culture medium. SCUBE1 therefore acts as an antagonist for long-range BMP signaling activity during brain development [20]. In contrast, when SCUBE1 is expressed in “signal-responding” cells, it forms a complex with BMP ligand and its receptors. SCUBE1 thereby acts as a BMP coreceptor to augment BMP signaling activity critical for protecting against kidney ischemia–reperfusion (I/R) injury or for primitive hematopoiesis [8, 49]. **B** Proposed model for adhesive function of SCUBE1 in platelet aggregation and thrombus formation. Under normal conditions, plasma SCUBE1 is expressed at low levels primarily by endothelial cells and platelets. In addition, SCUBE1 is stored in the α-granules of resting platelets. Upon pathological stimulation, activated platelets secrete large amounts of SCUBE1 into the circulation and high levels of SCUBE1 are also found on the platelet surface. The surface SCUBE1 on nearby activated platelets is trans-homophilically crosslinked by soluble SCUBE1, acting through its sticky EGF-like repeats to promote platelet agglutination and stabilize platelet plugs. Targeting the adhesive modules of SCUBE1 with a specific monoclonal antibody might be a potentially useful anti-thrombotic strategy [72, 86]. **C** SCUBE1-promoted BMP signaling protects against kidney ischemia/reperfusion (I/R) injury. I/R-induced SCUBE1 protein in proximal tubular epithelial cells serves as a BMP coreceptor to enable renoprotective BMP7 signaling, which stimulates epithelial repair and regeneration through proliferative, anti-apoptotic, and anti-inflammatory effects [49]. **D** Potential immunotherapy strategy and the pathological function of surface SCUBE1 in MLL-r leukemias. *Left: SCUBE1* is an immediate downstream target of the HOXA9/MEIS1 transcriptional regulatory complex, which is activated by MLL-fusion proteins like MLL-AF9 and is crucial for sustaining leukemic transition. Surface SCUBE1 functions as a FLT3 coreceptor in MLL-r leukemias, facilitating the interaction between FLT3 ligand and FLT3. This action increases downstream LYN-AKT activation (tyrosine phosphorylation) to enhance leukemic cell proliferation and survival, thereby promoting leukemogenesis. *Right:* An anti-SCUBE1 ADC conjugated to an antimitotic agent (MMAE) leads to significant cell killing specifically in MLL-AF9 leukemias. Thus, surface expression of SCUBE1 on MLL-r leukemia cells may be useful as a target for immunotherapy. *ADC* antibody–drug conjugate; *MMAE* monomethyl auristatin E. MLL-r, mixed-lineage leukemia gene-rearranged; *BMP* bone morphogenetic protein 1

Along the same lines, the CUB domain of SCUBE proteins can bind numerous growth factors and their cognate receptors to modulate the signaling strength of the growth factors. In particular, the SCUBE1 CUB domain can bind BMP2 [20], fms-like receptor tyrosine kinase 3 (FLT3) and FLT3 ligand [44]. Meanwhile, the CUB domains of SCUBE2 or SCUBE3 can interact with BMP2/4 [21], Sonic hedgehog (SHH) [45–47], Indian hedgehog (IHH) [45], and VEGF receptor 2 (VEGFR2) [23, 24] or FGF8 [9], FGF receptor 4 [9] and transforming growth factor β (TGF-β) type II receptor [48]. These interactions may serve to fine-tune the signaling intensities of many key cell signaling events (Table 2). Notably, the direction of effect by these SCUBE proteins (enhancing or suppressing the signaling strength) largely depends on whether the SCUBE protein is expressed in a signal-producing or signal-receiving cell. For instance, when BMP2 is co-expressed with a SCUBE1 C-terminal fragment containing the CR and CUB domains, secretion of BMP2 is markedly reduced, attenuating its long-range signaling activity [20, 22]. On the other hand, when overexpressed SCUBE1 is present on the surface of signal-receiving cells, it binds to BMP2/4/7 ligands and their cognate receptors, acting as a BMP coreceptor to increase BMP signaling activity (Fig. 3A) [8, 49, 50].

### Summary of SCUBE protein domain functions

In summary, data from cell culture experiments has shown that SCUBE molecules may be secreted or membrane-tethered N-glycoproteins, which are capable of forming homomeric or heteromeric complexes with other members [2, 5, 20, 21]. The *cis* or *trans*-oligomeric interactions of SCUBE proteins are mediated by EGF-like repeats and may be dependent on calcium binding [20, 22, 51]. In addition, a minimum of one triplet cbEGF_7-9_ is sufficient to drive self-adhesive interaction of SCUBE1 to an adjacent cell surface [20]. Although the spacer region and the CR motif alone are each sufficient for cell-surface tethering, cooperative actions of the two domains within full-length SCUBE1 are required for its secretion [26]. A molecular map revealed an essential polycationic area in the spacer, which most likely interacts electrostatically with anionic heparan sulfate proteoglycans on the cell surface to facilitate membrane attachment [26]. Furthermore, *N*-glycans of CR repeats greatly influence membrane localization, possibly due to lectin-mediated membrane presentation [26]. It is well known that the CUB domain, which is present in many developmentally regulated proteins, forms a β-barrel. In agreement with its previously reported functions in protein–protein or protein–carbohydrate interactions, we and others have clearly demonstrated that BMP proteins, hedgehog (HH) proteins, and many other growth factors or their receptors can bind to SCUBE proteins. These observations suggest that SCUBE proteins may serve as crucial regulators of growth factor signaling in diverse physiological and pathological processes (Table 2).

In the following sections, we elaborate further on the structural basis of SCUBE protein function and discuss the basic and clinical implications of each SCUBE family member. We also provide suggestions as to how current knowledge of SCUBE proteins may be translated to develop diagnostic, prognostic and therapeutic strategies for various human disorders.

## SCUBE1

### Regulation of gene expression and posttranslational control

SCUBE1 was identified as the first member of the SCUBE family. It was discovered in samples from human vascular ECs and the developing mouse urogenital ridge in the early 2000’s [1, 2]. In human samples, northern blot analysis revealed that *SCUBE1* mRNA is strongly expressed in highly vascularized tissues, such as liver, kidney and lung, with somewhat lower expression in brain, colon and spleen. Consistent with these findings, endothelial expression of SCUBE1 was demonstrated in monkey tissues by in situ hybridization, which showed that the gene is expressed in arterial, venous and microvascular ECs [2]. In addition, whole-mount or embryonic section in situ hybridization showed that mouse *Scube1* transcripts are expressed in the developing limb buds, nervous system, gonad, surface ectoderm, somites, and mesenchymal component of numerous tissues in the early head [1, 52]. For instance, *Scube1* was found to have strong, restricted expression in facial structures, such as the medial and lateral nasal processes, the maxilla and mandible, the caudal pharyngeal arches, and the dental papilla of both incisor and molar teeth, indicating a potential role during early craniofacial development (Table 3) [52]. Indeed, we reported that the first targeted *Scube1* knockout (KO) mice lacking functional CR and CUB domains exhibit an absent cranial vault and exencephaly. Moreover, we concluded that these phenotypes probably arise due to disruption of SCUBE1 antagonization of BMP family members via the CR and CUB domains (see below for further discussion) [20]. Additionally, *Scube1* mRNA is expressed throughout branching morphogenesis and in the urogenital system, suggesting that it plays a crucial role in the differentiation of metanephric mesenchymal cells during renal development [1, 53].

**Table 3:** Embryonic expression profiles of mouse *Scube* genes

Stage	*Scube1* [1, 52]	*Scube2* [3, 126]	*Scube3* [129, 162, 163, 165]
E8.5	Ventral forebrain		
E9.5	Ventral neural tube; head mesenchyme; mesenchyme of the first pharyngeal arch derivatives; limb buds	Forebrain: posterior telencephalon and the entire diencephalon, neuroepithelium of the optic cup; midbrain; hindbrain; spinal cord; rhombomere 5 and 6	Epithelium of early facial region (the first and second branchial arches, medial and lateral nasal process and otic vesicle); limb bud; neural tube
E10.5	Limb buds; infundibulum; derma myotome (somites); ventral neural tube; epithelium of Rathke’s pouch; trigeminal ganglion; lung bud; head mesenchyme; proximal mesenchyme of the second pharyngeal arch; early odontogenic mesenchyme of the maxillary and mandibular processes	Forebrain: telencephalic vesicle, optic cup; midbrain; hindbrain; spinal cord	Epithelium of the nasal pits and facial processes; midline of the frontonasal and maxillary processes; epithelium of otic vesicle and apical ectodermal ridge of the limb bud
E11.5	Neuroepithelium of the telencephalon; mesenchyme of the medial and lateral nasal processes; maxillary and mandibular process of the first pharyngeal arch; mesenchyme of the second and third pharyngeal arches	Forebrain: telencephalic vesicle, optic cup; midbrain; hindbrain; spinal cord; in the wall of 3rd ventricle; central canal	Epithelium of the fusing facial processes; otic vesicle; apical ectodermal ridge; ventral neural tube; ventral and dorsal dermomyotome
E12.5		Dorsal neural tube; dorsal diencephalon; wall of 4th ventricle; cartilage primordium of basioccipital bone and developing cervical vertebrae	Optic eminence; external nares; lip philtrum; vibrissae; developing incisor and molar tooth buds of the maxilla and mandible; epithelium of palatine processes
E13.5– E15.5	Mesenchyme of the nasal cavity; tongue root; periocular region; not In palatal shelves; mesenchyme surrounding the tooth buds; condensing mesenchyme of the dental papilla (E14.0)	Heart (ventricular tissue and major blood vessel); peripheral region of cartilaginous condensations of the axial skeleton; roof of 3^rd^ ventricle; nasal septum; tongue mesoderm; genioglossus muscle	Medial epithelium seam between the palatal shelves; developing teeth of the maxilla and mandible; early tooth bud epithelium, within the cap, staining was predominant in the enamel knot, internal and external enamel epithelium; at the bell stage, activity was seen throughout the differentiated cell populations within the enamel organ, including pre-ameloblasts and stellate reticulum; in the vibrissae, expression was seen in the dermal papilla, outer root sheath, and hair matrix; in the eye, expression was seen in the lens fibers, blood vessels of the hyaloid cavity, and in the neural layer of the retina; in E15.5, cartilaginous condensations of the peripheral skeleton, including the ribs, limbs and vertebra; the glomerular apparatus of the kidney

Genome mapping showed that *SCUBE1* is localized to human chromosome 22q13.2 and *Scube1* is found on mouse 15qE1 [1, 2]. There are no definitive reports of disease-associated *SCUBE1* mutations in humans, nor are there reports of N-ethyl-N-nitrosourea (ENU)-induced loss-of-function alleles causing *Scube1*-associated phenotypes in mice. One single nucleotide polymorphism (SNP) within *SCUBE1*, rs5759224 (intron genetic variant), was significantly associated with venous thromboembolism [54], and two de novo variants, NM_173050: exon 19: c.G2428A: p.E810K (damaging missense) and exon 9: c967 + 1G > A (likely gene disruption), are associated with obsessive–compulsive disorder [55]. Although our previous clinical investigation showed that acute myeloid leukemia (AML) patients with high levels of *SCUBE1* expression had worse overall and disease-free survival rates [44], we were unable to detect genetic gains or amplifications of *SCUBE1* in either AML or myelodysplastic syndrome cohorts (http://cbioportal.org). Instead, homozygous deletion of *SCUBE1* was identified in juvenile AML at a very low incidence (0.34%, 1 in 295 cases) [56]. Meanwhile, a natural variant, G398R, a SNP (dbSNP: rs129415), was detected in myelodysplastic syndrome at a low frequency of 0.39% (4 in 1026 cases). Further research will be necessary to determine whether chromosomal deletion of this *SCUBE1* SNP has any clinical impact.

*SCUBE1* expression in AML and primary AML samples has been examined, revealing that *SCUBE1* expression is upregulated in mixed-lineage leukemia gene-rearranged (MLL-r) AML cells, as compared to healthy hematopoietic stem and progenitor cells, peripheral blood cells, or leukemic cells devoid of the MLL gene rearrangement [44]. Despite the absence of a unique transcriptional regulator for *SCUBE1*, recent research has shown that in MLL-r leukemia, homeobox A9 (*HOXA9*) and Meis homeobox 1 (*MEIS1*) act together to upregulate *SCUBE1* [44]. HOXA9 is a homeobox protein with an important role in hematopoiesis and leukemogenesis. Its expression is primarily in less-differentiated cells, such as hematopoietic stem cells and progenitor cells, and is downregulated after lineage differentiation [57, 58]. The similarity of expression patterns for *HOXA9* and *SCUBE1* suggests that homeobox proteins, including HOXA9, might regulate *SCUBE1* during primitive hematopoiesis in mammals. In addition, our studies of renal ischemia–reperfusion (I/R) injury showed that SCUBE1 is a stress-responsive gene in ECs (positive for cluster of differentiation 31/CD31 and platelet endothelial cell adhesion molecule-1/PECAM-1) of the glomerulus and the peritubular capillary network [59] as well as proximal tubular epithelial cells [49]. The regulatory mechanisms underpinning this I/R-mediated induction of SCUBE1 remain unclear. Nevertheless, the 1.5 kb proximal promoter region of *SCUBE1* has more than five hypoxia-inducible factor (HIF)-binding sites (A/GCTGA), which are likely to be involved in the ischemia-induced SCUBE1 expression in renal ECs and tubular cells. Further investigations will be required to test whether these binding sites are indeed functional.

By substituting glutamine for asparagine at all *N*-linked glycosites, we created an *N*-glycan-deficient *SCUBE1*-ΔN construct. This construct was expressed in HEK-293T cells to explore the biological significance of *N*-glycosylation of SCUBE1 [26]. Both *SCUBE1*-wild type and *SCUBE1*-ΔN mutant cells produced comparable quantities of secreted protein, which implies that the ΔN mutant is structurally intact. However, due to the absence of *N*-glycans, the SCUBE1-ΔN mutant protein had a lower molecular mass, and it also exhibited poor cell-surface expression and regulatory activity as a BMP coreceptor [26]. Furthermore, mRNA injection of *scube1*-wild-type but not *scube1-ΔN* into *scube1*-knockdown zebrafish embryos could restore deficiencies in stem cell leukemia (*scl*) and GATA-binding factor 1 (*gata1*), two hematological and erythroid biomarkers. Thus, SCUBE1 function during hematopoiesis appears to depend on its *N*-glycan-mediated cell-surface expression and its ability to modulate BMP signaling in vivo [26].

### Developmental and physiological function

#### Brain development

Zebrafish genetic studies showed that the *ty97* allele (nonsense mutation in *scube2* encoding a truncated null protein lacking the CR and CUB domains) impairs HH and BMP signaling. These findings suggest that the CR and/or CUB domains are indispensable for Scube2 function [6, 7, 10]. To evaluate the biological function of the corresponding C-terminal portion of SCUBE1 in vivo, we generated a targeted disruption of mouse *Scube1*, which encodes a truncated protein lacking the CR and CUB domains [20]. This mutant allele was named *Scube1*^*Δcub*^, based on the lack of CUB immunoreactivity in the animal tissues [20]. Homozygous *Scube1*^*Δcub/Δcub*^ mice showed phenotypes of acrania, lack of brain tissue and death shortly after birth. Histological analysis of E12.5 embryos suggested that a brain defect between the diencephalon and myelencephalon led to exencephaly that was restricted to the midbrain, probably due to excessive neural tissue proliferation and everted cranial neural folds. In particular, hyperplasia was seen in the trigeminal and vestibulocochlear ganglia, with thickening of the forebrain neuroepithelium wall; the hindbrain neural tube was less impacted [20]. Overall, these brain malformation phenotypes are reminiscent of those in animals with BMP antagonist deficiencies, such as mice lacking Noggin and/or Chordin [60, 61]. These observations were in line with our molecular and biochemical findings that the CR and CUB domains are essential for SCUBE1 function in reducing long-range BMP signaling activity when co-expressed in the same cells [20]. Taken together, these studies strongly suggest that SCUBE1 plays a crucial function in the early phases of central nervous system development, presumably by regulating the activity of BMPs through its C-terminal CR and CUB domains (Fig. 3A).

#### Platelet function

Our previous immunohistochemistry results showed weak anti-SCUBE1 staining in microvascular ECs within a wide variety of vascular tissues, including the kidney, lung, gut and heart [62]. Yet, the immunoreactive signal was strongly restricted to the fibrin and platelet-rich regions of organized thrombi [62]. These findings are consistent with the notion that endothelial SCUBE1 might be released into the bloodstream, where it accumulates in thrombi formed by fibrin clots. SCUBE1 may also be expressed in platelets, similar to other endothelial membrane proteins like von Willebrand factor, P-selectin, and thrombomodulin [63–65]. Indeed, the expression of SCUBE1 was confirmed both at mRNA and protein levels in human platelets [62]. Further immunogold electron microscopy revealed that SCUBE1 is present in the membranes of α-granules of platelets, suggesting the organelles could be a potential location for SCUBE1 storage. SCUBE1 labeling was also seen in thin channels or open canaliculi, implying that SCUBE1 may be localized or linked to the membranes [62]. Furthermore, flow cytometry assays demonstrated that P-selectin and SCUBE1 were both expressed on the surface of thrombin-induced activated platelets [62].

The apparent molecular mass of SCUBE1 in resting platelets is about 130 kDa [20], which corresponds to the molecular mass of the full-length product [2]. In contrast, thrombus lysates reproducibly show a proteolytic fragment at ~ 95 kDa. It is known that the CUB domain of platelet-derived growth factor C or D must be proteolytically removed to reveal the physiologically active growth factor core domain [66–69]. Therefore, locally available fibrinolytic and coagulation proteases at a developing thrombus may perform a similar cleavage to release an active fragment from surface-bound or soluble SCUBE1. Although the precise nature of SCUBE1 processing is unclear, a proposed cleavage site (R_535_RXR_538_) for the furin-like protease [70] has been identified in the spacer region and may account for the serum-dependent proteolytic cleavage of SCUBE1 [71]. Consistently, a 95 kDa proteolytic SCUBE1 fragment is greatly increased in plasma from acute coronary syndrome patients who are experiencing plaque rupture/erosion and subsequent platelet activation; the same fragment is undetectable in healthy people [71]. A detailed analysis of the function of soluble SCUBE1 shows that it has a critical role in platelet agglutination and thrombus formation [62, 72]. Although soluble SCUBE1 alone cannot stimulate platelet activation, the SCUBE1 EGF-like repeat-containing protein fragment was shown to enhance ristocetin-induced platelet agglutination [62], which is analogous to platelet-subendothelial adhesion [73]. Since typical platelet aggregation stimulates the binding of fibrinogen to glycoprotein IIb–IIIa, the insensitivity of adenosine diphosphate or collagen-induced aggregation to additional SCUBE1 fragments further suggests a role for soluble SCUBE1 in platelet adhesion as opposed to platelet aggregation. Moreover, the EGF-like repeats of SCUBE1 on the surface of platelets likely mediate platelet self-adhesion [20]. For soluble SCUBE1, both its EGF-like repeats and its CUB domains have potential to interact with platelet endothelial aggregation receptor-1, a transmembrane receptor that contains EGF-like repeats and is expressed on the platelet surface, where it functions in platelet contact-induced activation [74] to mediate platelet agglutination [72].

#### Hematopoiesis

All vertebrates, including zebrafish, undergo primitive and definitive waves of hematopoiesis. In addition to its function in platelets, SCUBE1 has been shown to play a critical role in primitive hematopoiesis of zebrafish [8]. Knockdown of *scube1* in zebrafish embryos with antisense morpholino-oligonucleotides leads to the downregulation of marker genes for early primitive hematopoietic precursors [stem cell leukemia (*scl*)], erythroid lineage [GATA-binding factor 1 (*gata1*) and hemoglobin beta embryonic-1 (*hbbe1*)] as well as early and late myelomonocytic lineages [early: transcription factor PU. 1 (*pu.1*), and late: myeloperoxidase (*mpo*) and plastin-2 (*l-plastin*)] [8]. However, knockdown of *scube1* does not affect the expression of an early endothelial marker [fli-1 proto-oncogene (*fli1a*)], nor does it affect vascular morphogenesis. Importantly, genetic overexpression of *bmp* restores the expression of *scl* in the posterior lateral mesoderm during early primitive hematopoiesis in *scube1* morphants. This result suggests that scube1 may function as a Bmp coreceptor to promote Bmp signaling, as seen in biochemical and molecular studies. Combined, these findings in zebrafish suggest that *scube1* controls Bmp activity during zebrafish embryogenesis, which is essential for primitive hematopoiesis and places SCUBE1 at an early position in the regulatory hierarchy (Fig. 3A) [8].

### Pathological function

#### Acute coronary syndrome (ACS) and acute ischemic stroke (AIS)

Activated platelets are critical players in the initiation and progression of atherosclerosis [75], and SCUBE1 is a platelet granule protein that is exposed on the platelet surface upon activation [62]. Therefore, we performed immunostaining to examine the levels of SCUBE1 in the subendothelial matrices of atherosclerotic lesions, and found strong SCUBE1 staining in these areas [62]. The widespread, diffuse SCUBE1 staining pattern is suggestive of an extracellular localization of the protein in atherosclerotic plaques [62]. Moreover, SCUBE1 expression is not observed in macrophages or smooth muscle cells associated with atheromatous plaques, indicating that the SCUBE1 associated with atheroma may have an exogenous origin. Platelets and their granular contents, including von Willebrand factor, P-selectin and cluster of differentiation 40 (CD40) ligand, are closely associated with atherosclerotic plaques and play an important role in the development of disease [76–78]. Since SCUBE1 is generated by platelets and ECs, it might play a role in the pathophysiology of arteriosclerosis when it is deposited inside the subendothelial matrix of plaques. In addition, an adhesion assay showed that matrix-bound SCUBE1 can support platelet adhesion, and its soluble form can enhance ristocetin-induced platelet agglutination [62]. Together, these observations suggest that SCUBE1 might function as a key platelet-endothelial adhesion molecule in the pathophysiological development of cardiovascular disease.

According to a report from the World Health Organization, nearly 17.9 million people died from cardiovascular disease in 2019, accounting for 32% of all global deaths [79]. The most common three cardiovascular diseases are myocardial infarction, stroke and venous thromboembolism. In broad terms, thrombosis is defined as the formation of clots in arteries or veins. In the cases of myocardial infarction and ischemic stroke, the clots that form in an artery consist of platelets, while the clots in venous thromboembolism consist of red blood cells [80]. Platelet activation and aggregation are widely recognized as crucial reactions in arterial thrombosis and are responsible for ischemic complications in ACS or AIS. Since activated platelets can release soluble SCUBE1 into circulation [62], we hypothesized that the plasma level of SCUBE1 might serve as a reliable biomarker of platelet activation in acute atherothrombotic diseases. Indeed, our findings in clinical samples revealed that the plasma SCUBE1 concentration was high in ACS (n = 40) and AIS (n = 40) patients (median 205 and 95.1 ng/ml,* p* < 0.01), but it was virtually undetectable in healthy controls (n = 40) and people with chronic coronary artery disease (n = 83) [71]. At least four separate studies have included ELISA-based measurements of plasma SCUBE1 in healthy people (n = 40, 50, 50, 24), with average concentrations ranging from 30 to 50 ng/ml [71, 81–83]. The upregulation of plasma SCUBE1 is detectable early symptom onset (6 h) and remains so for at least three days. As such, the concentration of SCUBE1 in plasma can be considered an independent indicator of stroke severity based on the National Institute of Health Stroke Scale (β = 3.18, *p* < 0.001) [71]. In addition, small fragments of SCUBE1 have been identified in samples from ACS patients, which suggests that some proteolytic regulation/activation of plasma SCUBE1 may occur under pathological conditions [71]. Overall, these observations show that plasma SCUBE1 levels are markedly elevated in AIS and ACS patients but not coronary artery disease patients. As such, plasma SCUBE1 can be considered as a potential biomarker of platelet activation in acute thrombotic disease [71].

While the most effective reperfusion method for acute myocardial infarction is percutaneous coronary intervention with stenting, the intervention may lead to stent thrombosis involving activated platelets. Depending on the time delay after percutaneous coronary intervention, stent thrombosis can be classified as early (within 30 days of stenting) or late (within 1 year) stent thrombosis-elevation myocardial infarction (STEMI) [84]. Although stent thrombosis is an uncommon event, it is becoming a significant global health issue due to the increasing number of percutaneous coronary interventions undertaken around the world. Strikingly, the fatality in STEMI can be up to 18% [85], so accurate predictors for STEMI are urgently needed. Bolayir et al. found that the plasma level of SCUBE1 may be a promising biomarker for both early and late STEMI [81, 82]. In particular, the authors showed that the plasma level of SCUBE1 in patients with STEMI can reach ~ 100 ng/ml, and it can be considered an independent prognostic factor. Furthermore, the plasma SCUBE1 level was shown to be positively correlated with soluble CD40 ligand (sCD40L) level in hemodialysis patients, signaling myocardial infarction.

#### Platelet activation and thrombosis

To further define the precise role of plasma SCUBE1 in vivo, we generated a second version of mutant (Δ) mice that lack soluble but retain membrane-bound SCUBE1. To generate these mice, we removed the genetic sequences encoding a portion of the spacer region and the entire region encoding the three CR repeats. However, the C-terminal CUB domain was still transcribed in-frame in the mutant Δ/Δ mouse platelets [72]. In the plasma SCUBE1-depleted Δ/Δ mice, major platelet receptor expression is unaffected, yet Δ/Δ platelet-rich plasma exhibits abnormal platelet aggregation in response to adenosine diphosphate and collagen. Notably, recombinant SCUBE1 protein treatment improves platelet aggregation in wild-type platelet-rich plasma, and it also restores platelet aggregation in Δ/Δ platelet-rich plasma. The deficit in active plasma SCUBE1 attenuates arterial thrombosis in mice and provides protection from lethal thromboembolism after collagen-epinephrine treatment. Collectively, these observations suggest that plasma SCUBE1 contributes to platelet aggregation by joining adjacent activated platelets during thrombosis. Hence, it is possible that inhibition of soluble SCUBE1 could exert antithrombic actions (Fig. 3B) (see below for further discussion).

Our studies to this point had clearly shown that soluble SCUBE1 is not only a potential biomarker of platelet activation [71], but it is also an active participant in thrombosis [20, 62, 72]. However, we still did not know whether the adhesive EGF-like repeat module is crucial for its activity, and we also did not know the specific contributions of SCUBE1 produced in ECs and platelets during thrombosis. To address these questions, we generated a third version of mutant (Δ2) mice, which lack the entire EGF-like repeat module, and we used these mice to further evaluate the functional significance of this module during in vivo thrombogenesis [86]. The platelet-rich plasma from Δ2 mice exhibits a profound deficiency in platelet aggregation, as induced by a wide range of agonists [adenosine diphosphate (ADP), collagen, thrombin agonist protease-activated receptor 4 (PAR-4) peptide, and thromboxane A2 analogue U46619]. Moreover, genetic deletion of EGF-like repeats also prevents deadly thromboembolism in Δ2 mice and reduces arterial thrombosis [86]. A flow chamber assay was also used to demonstrate that blocking antibodies against the EGF-like repeats in whole blood from wild-type mice can significantly reduce platelet deposition and thrombus formation on collagen-coated surfaces under arterial shear rates. By performing reciprocal bone-marrow transplantation in wild-type and Δ2 mice, we generated animals that only produce SCUBE1 in either ECs or platelets. The animals that only produce SCUBE1 in ECs exhibit a prolonged time to carotid arterial thrombosis after ferric chloride administration, which suggests that platelet-derived SCUBE1 is a crucial factor in arterial thrombosis. This effect is likely mediated by the adhesive EGF-like repeats in platelet-derived SCUBE1, so targeting the adhesive motifs of SCUBE1 may be a potentially useful anti-thrombotic strategy (Fig. 3B).

sCD40L is a tumor necrosis factor expressed in several immune-specific cells, including platelets [87]. Like SCUBE1, sCD40L is stored in platelet α-granules, and it is released upon activation. Furthermore, sCD40L is a biomarker for platelet activation [88], ACS [89] and sepsis [90]. Plasma sCD40L and SCUBE1 levels are correlated in patients with cardiovascular disease [83, 91–93] and in in patients with hypertension [83, 92]. According to the ambulatory blood-pressure monitoring data classifications, a ≥ 10% decrease in night-time blood pressure is called “dipper” hypertension, and a < 10% decrease is “non-dipper” hypertension [83, 94]. Guzel et al*.* analyzed SCUBE1 plasma levels in dipper and non-dipper hypertensive patients [83]. Although the plasma SCUBE1 level is elevated in both classes of hypertensive patients, it is significantly higher in non-dipper patients than dipper patients. However, the level of sCD40L is not significantly different when comparing between the two classes. Since non-dipper hypertension is considered to be more severe than dipper hypertension, SCUBE1 can be used to differentiate between the classes and is considered a biomarker for severe hypertension.

#### Pulmonary hypertension

Pulmonary hypertension is high blood pressure in pulmonary arteries, which supply the lungs. The condition is characterized by a mean pulmonary artery pressure of ≥ 25 mmHg with a normal pulmonary artery wedge pressure of ≤ 15 mmHg, in addition to increased pulmonary vascular resistance > 240 dyn·s·m^−5^ [95]. Pulmonary hypertension affects 1% of all adults globally and has a 5-year mortality rate of approximately 40% [95, 96]. This chronic and progressive disease can lead to heart failure and death when left untreated. Pathogenic mutations in the BMP receptor 2 (BMPR2) can cause one of the most severe types of pulmonary hypertension. In line with our studies implicating SCUBE1 in BMP signaling [8, 20, 49], it was shown that SCUBE1 is downregulated in pulmonary arterial hypertension (PAH) with BMPR2 mutation [97]. The study further showed that the SCUBE1 expression level is decreased in ECs derived from human induced pluripotent stem cell ECs with BMPR2 mutation as compared with wild-type controls. Induction of hypoxia or treatment with the inflammatory cytokine interleukin-1β were used to trigger PAH. In BMPR2-mutated cells, these treatments reduce SCUBE1 expression at the transcript and protein levels and also reduce the secreted protein level, whereas most examined EC-specific genes (e.g., VEGF and endothelial nitric oxide synthase) are upregulated [97]. Moreover, SCUBE1 was shown to be downregulated via HIF-1α in this context. The study showed that SCUBE1 acts by regulating Smad1/5/8 signaling, which is relevant to BMPR2 but not TGF-β–related SMAD2/3 signaling. Consistent with the cell-based results, the authors also found reduced SCUBE1 levels in plasma and lung tissue both in the animal model of PAH and in patients with PAH. Therefore, SCUBE1 downregulation in the context of pulmonary hypertension could be a specific biomarker of disease.

#### Kidney injury

As mentioned in previous sections, zebrafish Scube1 forms a complex with Bmp ligands and its receptors to act a BMP coreceptor and augment BMP signal activity [8]. Nevertheless, it remained unknown if mammalian SCUBE1 can bind to BMP7 and enhance activation of its downstream signaling pathway. This activity would be relevant to kidney injury, as BMP7 is a preventive factor for renal I/R damage [98, 99], which may also facilitate renal tubular cells proliferation and healing after I/R injury. Recently, we demonstrated that proximal tubular cells exhibit I/R-induced upregulation of SCUBE1 expression together with renoprotective BMP7 expression. Further molecular and pharmacological studies showed that SCUBE1 promotes BMP7 signaling by acting as a BMP coreceptor, directly binding to BMP7 and its receptors [49]. For this study, we used *Scube1*-deletion mutant (Δ2) mice to clarify the pathophysiological role of SCUBE1 following kidney I/R damage. The Δ2 mice show more severe histopathologic features than wild-type littermates, in terms of tubular necrosis and dilation and loss of brush border. The Δ2 mice also have increased inflammatory response, including neutrophil infiltration, and induction of tumor necrosis factor-α, monocyte chemoattractant protein-1 and interleukin-6, during the resolution phase of I/R damage [49]. Furthermore, the Δ2 mice show reduced BMP signaling strength (in terms of phosphorylated Smad1/5/8) together with increased apoptosis and decreased proliferation of kidney tubular cells compared to controls [49]. Together, these results reveal a novel cell-autonomous function of I/R-induced SCUBE1 protein in proximal tubules. We can therefore conclude that surface SCUBE1 functions as a coreceptor to promote BMP7-mediated proliferative, anti-inflammatory, and anti-apoptotic effects to protect from injury and promote epithelial repair and regeneration (Fig. 3C) [49]. However, another paracrine mode of action has also been proposed, whereby SCUBE1 is secreted from peritubular capillary ECs after I/R injury and mediates renal remodeling and cellular replacement by increasing tubular cell proliferation [59]. Nevertheless, the SCUBE1-induced signaling pathway that mediates its reparative paracrine effects on adjacent kidney tubules is still largely unknown [59]. Most importantly, it remains an open question whether stress-induced expression of endothelial or epithelial SCUBE1 may play some role in pathophysiological conditions other than the kidney.

#### Acute myeloid leukemia (AML)

Although the SCUBE1 plasma level is high in the context of some cancers, such as renal and breast cancer, the exact functions of SCUBE1 in cancer cells remain largely unknown and uninvestigated. In AML, SCUBE1 is known to be abundantly expressed on the MLL-r AML cell membrane and predicts a poor prognosis in de novo AML, and we recently showed that the *SCUBE1* gene is a direct target of HOXA9/MEIS1 [44, 100]. These findings raise the possibility that SCUBE1 may have oncogenic properties. For instance, knockdown of* SCUBE1* in human MLL-r AML cells reduces leukemic cell engraftment in an in vivo model, and it lowers cell viability and enhances apoptosis in vitro. Along the same lines, ablation of *Scube1* in murine hematopoietic progenitor cells greatly reduce the potential for oncogenic MLL-AF9-mediated leukemic initiation, even though SCUBE1 overexpression was insufficient to promote neoplastic transformation. We also assessed whether *Scube1* plays a role in sustaining MLL-r AML development in vivo using the *Scube1* conditional knockout mouse model [44]. The survival of mice receiving MLL-AF9 leukemia cells is dramatically improved by tamoxifen-induced deletion of *Scube1*. Collectively, our observations strongly imply that SCUBE1 plays important roles in the development and persistence of MLL-r leukemia [44].

In an effort to further clarify the molecular pathways affected by membrane SCUBE1 during leukemogenesis, we performed unbiased proteomic proximity labeling and mass spectrometry analysis to identify membrane proteins in close proximity to surface SCUBE1 [44]. FLT3 and its direct signaling component lck/yes-related novel protein tyrosine kinase (LYN, a non-receptor protein tyrosine kinase) are both associated with cell-surface SCUBE1 in MLL-r AML cells. Further gain- and loss-of-function, biochemical, and molecular studies demonstrated that the membrane-tethered SCUBE1 binds to extracellular ligand-binding domains of FLT3, which promotes signaling along the FLT3-LYN axis in order to enhance leukemic growth and survival signals [44]. Thus, our results showed that aberrantly upregulated SCUBE1 binds to the HOXA9/MEIS1 target FLT3, stabilizing FLT3 protein and promoting its activation. This mechanism appears to represent a feed-forward mechanism that serves to enhance the FLT3–LYN signaling and also reinforce the signaling network downstream of HOXA9 and MEIS1 during leukemogenesis. Overall, this study shed light on critical pathological roles of SCUBE1, a newly identified transcriptional target of HOXA9/MEIS1, in MLL-r AML initiation and maintenance (Fig. 3D).

### Potential translational applications and therapeutic strategies

As stated above, SCUBE1 concentrations are low in plasma derived from PAH animal models as well as patients with PAH [97]. In addition, plasma SCUBE1 concentrations are closely associated with hemodynamic markers of disease severity. Thus, SCUBE1 is implicated as a contributor to BMPR2-related PAH pathogenesis, which suggests that it may be a relevant target for therapeutics, diagnosis, and/or prognosis of the disease [97].

Given the susceptibility of Δ2 mutants after I/R injury and the novel BMP7-enhancing effects of upregulated SCUBE1 in response to I/R stress [49, 59], targeting SCUBE1 might be a feasible treatment strategy for acute kidney injury. This could be accomplished by infusion of recombinant SCUBE1 protein, gene therapy to induce targeted renal SCUBE1 expression, or injection of compounds that can effectively and specifically upregulate renal SCUBE1 protein. Moreover, plasma SCUBE1 level is greatly increased due to secretion from platelets and/or ECs in pathologic conditions, making it a possible biomarker of platelet activation in the context of ACS, AIS, hemodialysis or hypertension. Similarly, injured or regenerating tubular cells may release membrane-tethered SCUBE1 into the urine, which may have diagnostic or prognostic utility for acute kidney injury. Clinical studies will be required to test the utility of urinary SCUBE1 as a biomarker and to assess whether supplementation of exogenous SCUBE1 protein could provide therapeutic benefit for kidney disease.

Because our cumulative in vitro/ex vivo platelet binding/aggregation assays [20, 62] and in vivo genetic ablation studies [72, 86] suggested that the EGF-like repeats of SCUBE1 are essential for thrombogenesis, we tested whether blocking the EGF-like repeats of SCUBE1 might prevent thrombosis. To investigate the effects of particular antibodies targeting EGF-like repeats of SCUBE1 on platelet adhesion and thrombus formation, we performed proof-of-concept experiments using a flow chamber. Two different monoclonal antibodies, mAb #2 and mAb #7, were respectively raised against EGF_1-3_ or EGF_4-6_ of SCUBE1. To assess the formation of thrombi on a collagen-coated microfluidic device, whole blood from wild-type mice pre-incubated with isotype control antibody, anti-SCUBE1 mAb #2, mAb #7 or anti-CUB was perfused through the flow chamber at a high shear rate of 1000 s^−1^ [86]. We found that treatment with mAb #2 or mAb #7 only slightly increases initial platelet adhesion, but it significantly decreases subsequent platelet recruitment and thrombus formation. In contrast, control IgG and anti-CUB mAb seem to have no impact on platelet adhesion or thrombus formation, indicating that the specific targeting of EGF-like repeats by mAb #2 and mAb #7 is crucial for the effect [86]. Furthermore, mAb #7 blocks ristocetin-induced platelet agglutination [72] and protects wild-type mice against fatal thromboembolism triggered by collagen and epinephrine (60% survival of treated mice, compared with 0% survival in saline and isotype control groups). mAb #7 also attenuated arterial thrombosis formation due to ferric chloride injury [72]. Importantly, mice injected with anti-thrombotic SCUBE1 mAb #2 or #7 exhibit no evidence of prolonged bleeding times [72, 86]. Collectively, these findings demonstrate the critical functions of EGF-like domains in the recruitment and deposition of platelets during aggregation. Antibodies targeting SCUBE1 EGF-like repeats may therefore be promising tools for the prevention of thrombosis (Fig. 3B).

Once an antibody binds to a cell-surface target, its internalization and transport to lysosomes allows an antibody–drug conjugate (ADC) to execute its killing effect, so long as the cytotoxic payload can be released from the lysosomes [101]. We therefore generated an anti-SCUBE1 mAb #1 (a different clone from the above-mentioned antithrombotic mAb #2 and mAb #7) that could be rapidly bound, efficiently endocytosed to lysosomes and degraded within 24 h. This timeline suggested that the mAb could be efficiently internalized and directed to lysosomes [44]. We then produced a SCUBE1-targeting ADC by linking mAb #1 to an enzymatically cleavable valine-citrulline linker and an anti-microtubule cytotoxic agent monomethyl auristatin E (MMAE). Importantly, the ADC (anti-SCUBE1-valine-citrulline-monomethyl auristatin E) retains binding affinity similar to the parental antibody [44]. In addition, the ADC can selectively kill SCUBE1-expressing MLL-r leukemia cells (THP-1 or NOMO-1) but not SCUBE1-negative KG-1a or K562 leukemic cells in vitro, and it can suppress MLL-r leukemia growth in a xenograft model [44]. The ADC-receiving mice also show no signs of antigen-independent toxicity, as indicated by weight loss. Collectively, these findings demonstrate the selectivity of the ADC and support the idea that surface SCUBE1 has potential as a therapeutic target and MLL-r-specific biomarker (Fig. 3D).

## SCUBE2

### Regulation of gene expression and posttranslational control

Research on SCUBE2 has been largely driven by findings from zebrafish genetic studies [6, 7, 10] and human breast cancer genomic studies [102–106]. Notably, a series of reports using a positional cloning approach showed that the zebrafish homologue of *scube2* is required for HH signaling during the development of the ventral spinal cord, dorsal aorta, and slow muscle [6, 7, 10]. Meanwhile, human *SCUBE2* has been implicated in breast cancer biology. As such, *SCUBE2* was the only overlapping breast cancer-associated gene identified in a microarray gene expression profiling study [102] involving a cross-platform comparison of gene lists that define a molecular signature for breast cancer (70 genes) [103, 104] and recurrence score models [105, 106] for predicting the prognosis of breast cancers.

The zebrafish genetic analyses suggest that Scube2 acts upstream of the HH response mechanism [6, 7, 10] and that it can attenuate Bmp activity [7]. Of note, a nonsense mutation in the *ty97* allele encodes a truncated Scube2 product lacking the CR and CUB domains, which suggests that these domains are indispensable for Scube2 function [6, 7, 10]. In line with these genetic studies, molecular and biochemical experiments showed that SCUBE2 can positively regulate SHH signaling via separate modes of action on signal-producing and signal-receiving cells. In the first mechanism, the soluble form of SCUBE2 facilitates the release and solubilization of dual-lipidated SHH ligand by acting as a chaperone for dual-lipidated HH or as an enhancer of HH shedding. In the dual-lipidated HH ligand, a palmityl moiety is attached at the N-terminus of HH by palmityl transferase [107] and a cholesteryl residue is linked at the C-terminus by autocatalysis [108]; this form of HH is mainly involved in long-range signaling [46, 47, 109–111]. In the second mechanism, membrane-bound SCUBE2 within lipid rafts (cholesterol-concentrated membrane microdomains enriched with cholesterol-modified HH ligand) can bind, concentrate and promote the presentation of SHH to the patched homolog 1 receptor in signal-responding cells [4]. Therefore, SCUBE2 positively modulates HH signaling activity, either by promoting the release of HH ligand from signal-producing cells or by enhancing cellular signaling in the signal-receiving cells. In addition, a zebrafish genetic study showed that Scube2 can attenuate Bmp activity [7]. Similarly, our clinical, biochemical and molecular studies showed that in the context of breast cancer, high SCUBE2 expression suppresses breast tumor cell proliferation and is associated with a favorable prognosis for invasive breast cancer, likely because the CR and CUB domains directly bind BMP proteins and antagonize long-range BMP signaling [112].

A commercially available cDNA clone for human SCUBE2 represents a splice variant lacking the ninth EGF-like repeat and a portion of the spacer region [2]. Therefore, we swapped in a cDNA fragment to correct the defective region in this clone [4]. The resulting human SCUBE2 full-length cDNA encodes a polypeptide containing a fully organized protein domain structure in line with zebrafish *scube2* (Table 1) [4]. Interestingly, a major difference between SCUBE2 and SCUBE1 is that SCUBE2 carries an extra 30 amino acid residues (encoded by a single exon) between the fifth and sixth EGF-like repeats (Fig. 1A) [4]. This added stretch contains a potential N-linked glycosylation site and also a minimal furin cleavage site (R_276_XXR_279_), which can allow for release of an N-terminal cleaved fragment containing the EGF_1–5_ repeats from the rest of the protein [4]. Of note, this furin consensus site was ablated to improve the yield of full-length SCUBE2 protein in a biochemical study on SHH signaling [113]. Currently, the biological relevance of this extra stretch of 30 amino acids in SCUBE2 remains unknown, but it can be postulated that proteolytic cleavage might represent a regulatory mechanism that could be critical for SCUBE2 function. Analogously, upregulated SCUBE2 in breast cancer primary tumor cells can be proteolytically cleaved by matrix metalloproteinase 2 (MMP-2) to separate the C-terminal CR and CUB domains from the N-terminal EGF-like repeats and the spacer region [112]. It was shown that only the C-terminal CR and CUB domains but not the full-length protein or the *ty97* mutant lacking the CR and CUB domains can potently suppress the secretion of mature BMP2 into conditioned medium [112]. Consistent with these data, overexpressed full-length SCUBE2 and *ty97*-encoded protein are secreted into culture medium and properly targeted to the cell surface, while the fragment containing only C-terminal CR and CUB domains is defective in membrane association and secretion [112]. Intriguingly, the C-terminal fragment is subject to regulatory processing via MMP-2. Since the fragment is incapable of membrane binding or secretion, it confines BMP ligands within the signal-producing cells, thus preventing BMP secretion and long-range signaling activity [112]. The findings from our breast cancer studies might therefore partly explain why the zebrafish Scube2 can attenuate Bmp in a permissive manner [7]. The anti-Bmp activity of zebrafish Scube2 may depend on the concurrent presence of some as yet unidentified cofactor, although MMP-2 is a likely candidate. Further investigations will be needed to test this possibility.

Human *SCUBE2* and mouse *Scube2* are respectively located at chromosome 11p15.4 and 7qE3. Like *SCUBE1*, no germline *SCUBE2* mutations are directly associated with human diseases, and no ENU-induced phenotypes are associated with mutations in mouse *Scube2*. Nevertheless, a genome-wide association study on aberrant plasma β_2_-glycoprotein I levels identified an intronic SNP (rs963167) from *SCUBE2* that is significantly associated with the β_2_-glycoprotein I phenotype [114]. β_2_-Glycoprotein I levels are associated with autoimmune diseases, hemostasis, atherogenesis and angiogenesis. However, the authors were unable to detect a significant association between the identified *SCUBE2* variant and coronary artery disease in an in silico follow-up study [114]. Although the genome-wide association study provided evidence that *SCUBE2* is a genetic determinant of plasma β_2_-glycoprotein I level, further investigations will be required to elucidate the putative role of *SCUBE2* in β_2_-glycoprotein I-associated cardiovascular and autoimmune diseases. In addition, a recent study using targeted sequencing of previously identified linkage regions revealed a missense variant (rs61751342) within the chromosome 11 quantitative trait locus that co-segregates with carotid plaque [115]. The rs61751342 missense variant is located in the DENN domain-containing 2B gene (*DENND2B*) and is an expression quantitative trait locus for *SCUBE2* in the atrial appendage (*p* = 3.5 $\times$ 10^–5^, GTEx Consortium) [115].

### Developmental and physiological function

#### Regulation of the HH signal pathway

Zebrafish is the first in vivo model in which Scube2 activity was found to be involved in the control of the HH signal pathway. Furthermore, this regulatory mechanism was observed during development of slow muscle, the ventral spinal cord, and the dorsal aorta [6, 7, 10, 116]. In addition, a zebrafish genetic and biochemical study showed that Scube2 is also involved in maintaining the structure and function of the notochord, a transiently existing tissue that exerts both mechanical and signaling cues during vertebrate embryonic development [117]. In particular, Scube2 interacts with Emilin3 in the extracellular matrix of the notochord sheath, which inhibits the regulatory activity of Scube2 on release of HH ligands by notochord cells [117]. Thus, Emilin3 ensures proper notochord patterning by supporting the integrity and function of the notochord sheath [117].

The HH signaling pathway was first identified in *Drosophila* and has been found to be highly conserved in vertebrates. Although there is only one HH in the fruit fly, the mammalian HH family includes three ligand proteins, SHH, IHH and desert hedgehog (DHH), all of which are involved in morphogenesis and organogenesis [118, 119]. During prenatal skeletal development, IHH is mostly expressed in prehypertrophic chondrocytes, and it serves to regulate chondrocyte differentiation and proliferation, guiding the differentiation of perichondrial mesenchymal cells into osteoblasts [120–124]. In the postnatal period, chondrocyte-derived IHH plays a crucial role in preserving the growth plate, trabecular bone development, and skeletal growth [125]. Using whole-mount and section in situ hybridization, the mouse *Scube2* transcript was found to be expressed in the embryonic neuroectoderm, the developing face, heart, vasculature, and the perichondrium of endochondral skeletal structures, such as developing ribs, lumbar vertebrae and long bones of limbs (Table 3) [3, 126]. The primitive mesenchymal cells that constitute the perichondrium surrounding the growth-plate cartilage develop into osteoblasts that later form the cortical and trabecular bones [127]. Nonetheless, it remains unknown whether mammalian *SCUBE2* participates in endochondral bone formation or other developmental processes, as has been observed for zebrafish *scube2*.

#### Endochondral bone formation

**Fig. 4:**
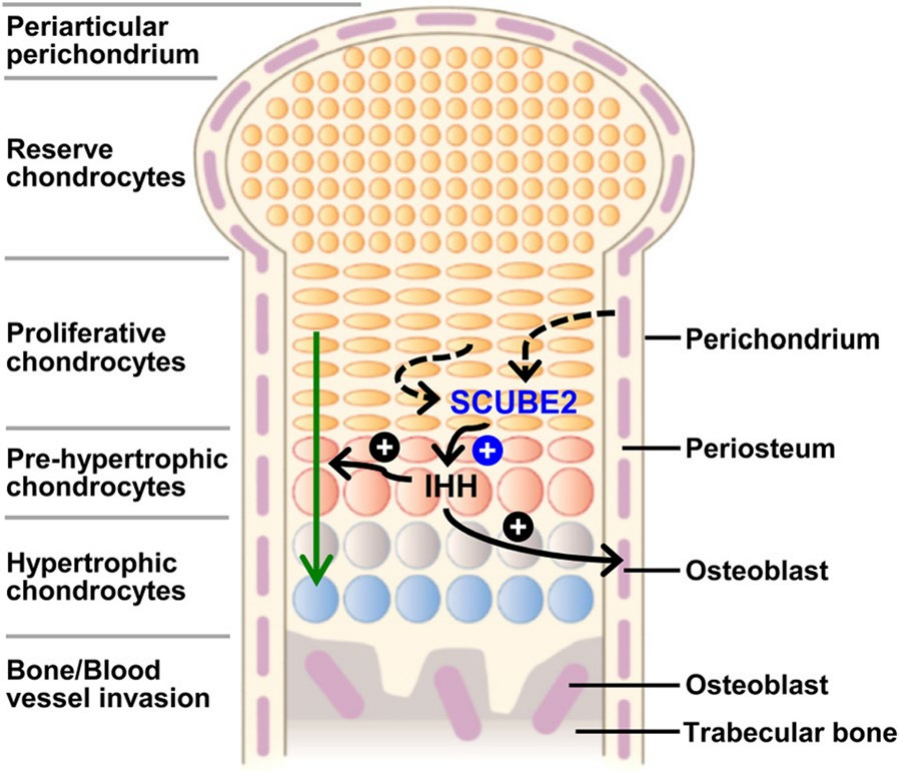
SCUBE2 is essential for controlling IHH signaling during endochondral ossification. SCUBE2 protein is expressed in peri-chondrial cells and may be secreted (dashed line with arrow). This secreted SCUBE2 could modulate IHH signals derived from pre-hypertrophic chondrocytes (solid line with arrow) by promoting the release and mobilization of IHH, which drives differentiation into osteoblasts during endochondral bone formation. Green arrow indicates the differentiation direction toward hypertrophic chondrocytes within endochondral bone. *IHH* Indian hedgehog

Our study demonstrated that soluble SCUBE2 is more effective than SCUBE1 or SCUBE3 at modulating IHH signaling in vitro [45]. Further experiments in mice showed that Scube2 plays a role in osteogenesis by facilitating *Ihh*-stimulated osteoblast formation from mesenchymal progenitor cells. In line with the these findings in vitro and the role of *Ihh* in orchestrating skeletogenesis, we further found that genetic knockout of *Scube2* (deletion of all coding exons) results in poor *Ihh*-mediated chondrocyte differentiation and proliferation, along with defects in osteoblast differentiation and deficient endochondral bone formation in bone-marrow mesenchymal stromal cell cultures [45]. Together, these findings suggest that *Scube2* is an essential determinant of *Ihh*-dependent endochondral bone formation. Particularly, SCUBE2 protein produced in perichondral cells may influence prehypertrophic chondrocyte-derived IHH signaling by facilitating the release and mobilization of IHH to promote differentiation of osteoblasts during endochondral bone formation (Fig. 4).

#### Neovascularization

**Fig. 5:**
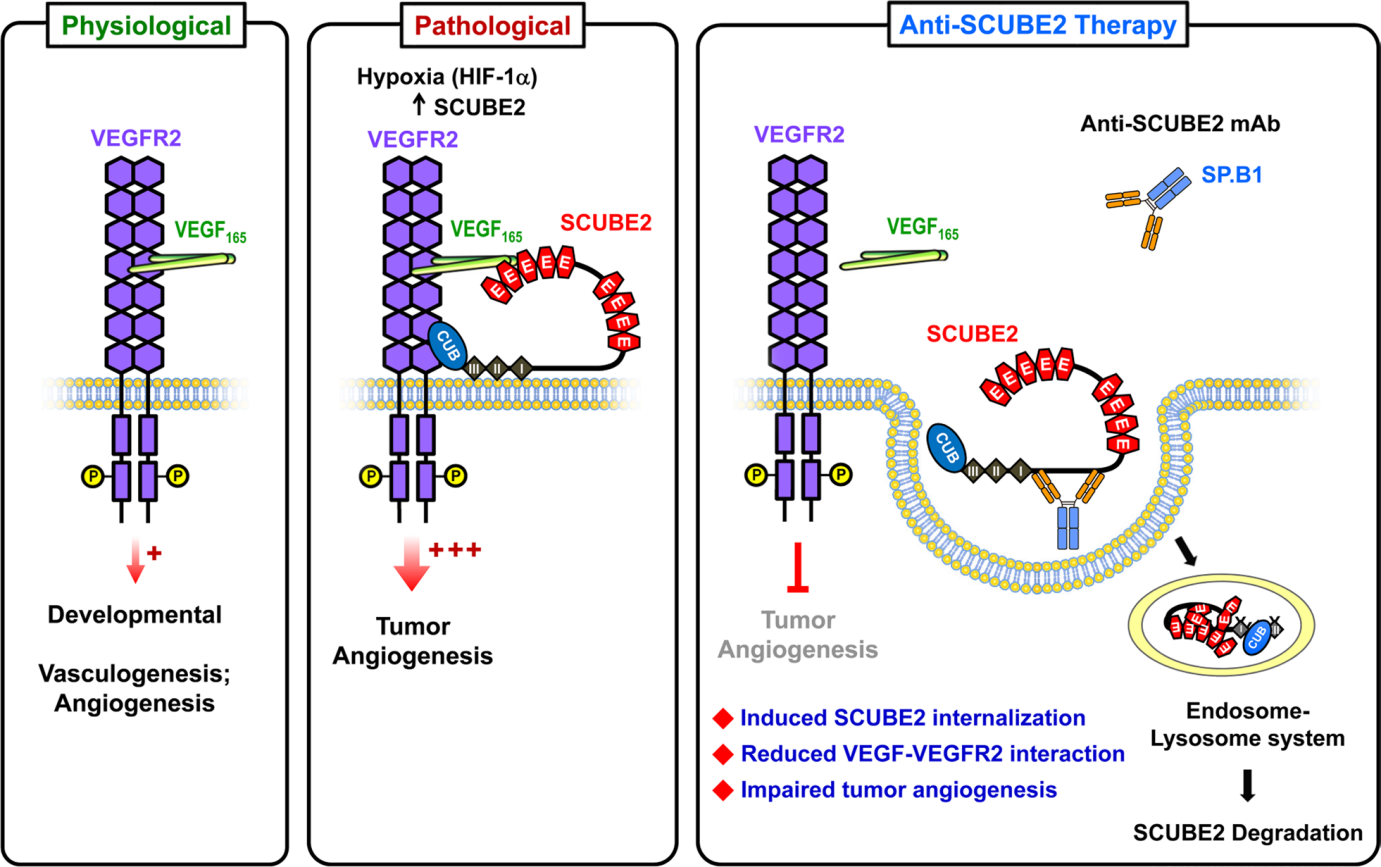
Physiological and pathological roles of endothelial SCUBE2 in modulating VEGF signaling during angiogenesis and potential immunotherapy approach. VEGF-stimulated VEGFR2 signaling is essential for angiogenesis and vasculogenesis under physiological conditions, such as during embryonic development (left panel). However, under pathological circumstances (e.g., intratumor hypoxia), hypoxia-inducible HIF-1 upregulates SCUBE2. The SCUBE2 protein localizes on the tumor EC cell surface, where it functions as a coreceptor with VEGFR2 to facilitate VEGF binding and enhance its downstream signaling. Thus, SCUBE2 promotes VEGF-induced tumor angiogenesis [23] (middle panel). SCUBE2 is internalized into the endosome-lysosomal protein degradation pathway when SP.B1 mAb attaches to EC-surface SCUBE2. This internalization decreases the VEGF-VEGFR2 association and prevents tumor angiogenesis (right panel). EC, endothelial cell. *VEGF* vascular endothelial growth factor; *VEGFR2* VEGF receptor 2

VEGF is an essential angiogenic factor that drives proangiogenic signaling by binding to VEGFR2, which is also subject to regulation by VEGFR2 coreceptors such as neurophilins [128]. Since vascular EC-derived SCUBE2 is known to be a peripheral membrane protein that binds and functions as a coreceptor for other signaling receptors [4, 45, 51], we conducted biochemical and molecular studies that revealed a role for SCUBE2 in the VEGF/VEGFR2 signaling pathway [23]. In our study, we found that SCUBE2 overexpression increases VEGF-induced EC proliferation and capillary-like network formation on Matrigel, while its knockdown has the opposite effect. We also showed that similar to VEGF, the transcriptional and translational levels of endothelial SCUBE2 are also upregulated by HIF-1α. Consistently, recovery of blood flow after hind-limb ischemia and VEGF-induced neovascularization in implanted Matrigel plugs are impaired in EC-specific *Scube2*-knockout mice. Co-immunoprecipitation and binding assays further showed that VEGF and VEGFR2 both bind to SCUBE2, suggesting that SCUBE2 acts as a coreceptor to facilitate VEGF binding and augment VEGFR2 signal activity (Fig. 5). SCUBE2 deficiency (knockout or knockdown) suppresses VEGF-mediated activation of proliferation and proangiogenic signals, while SCUBE2 overexpression enhances the signaling readouts, including phosphorylation of VEGFR2 and activation of mitogen-activated protein kinase (MAPK)/AKT.

**Fig. 6:**
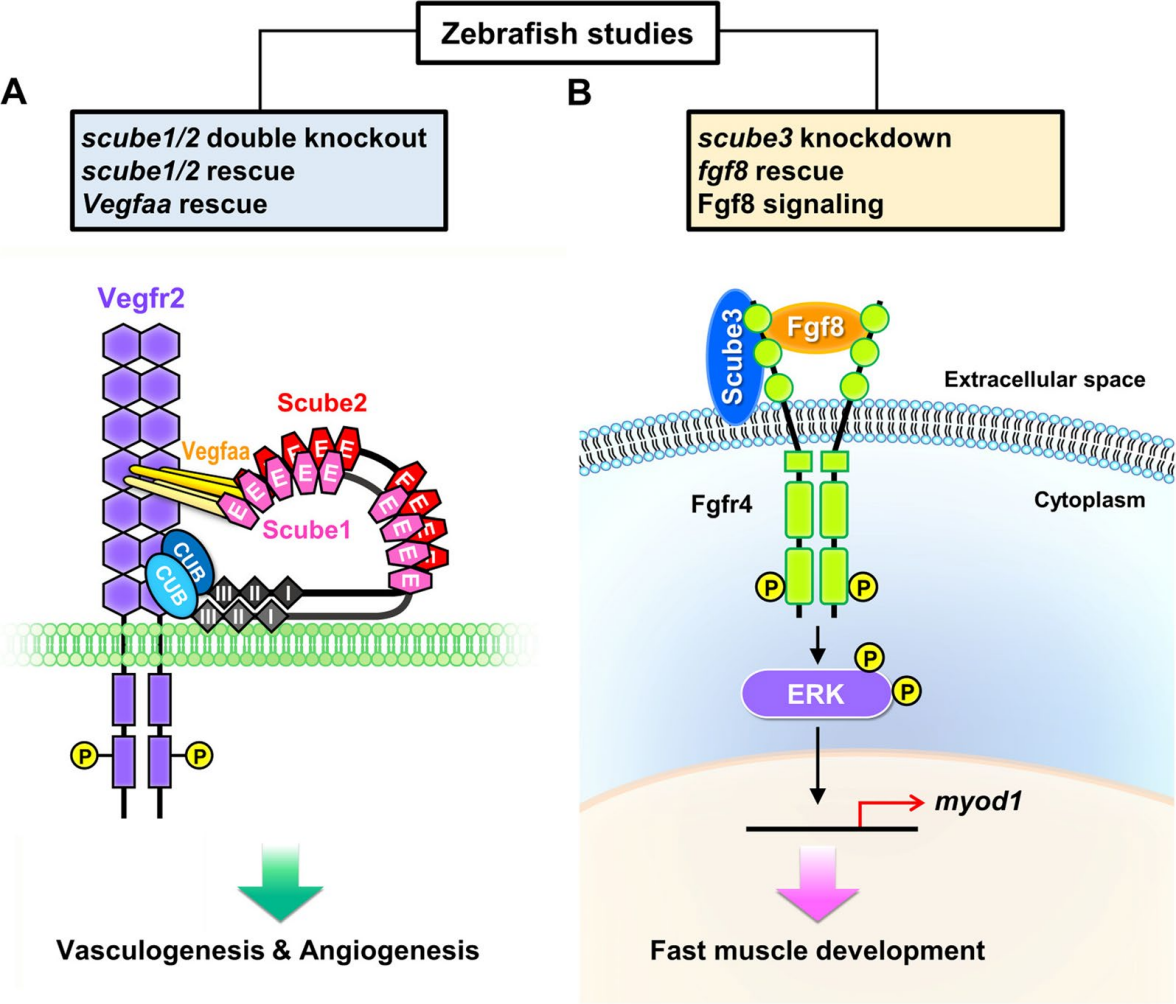
Functional studies of SCUBE family using the zebrafish model. **A** Scube1 and Scube2 from zebrafish endothelial cells work together to support Vegfa signaling during embryonic vascularization. Endothelial Scube1 and Scube2 form a complex under physiological conditions, such as during the development of embryonic vessels. This complex may promote Vegf-induced Vegfr2 phosphorylation and its downstream signaling for proper vasculogenesis and angiogenesis [130]. **B** Zebrafish Scube3 acts as an Fgf coreceptor during fast muscle fiber development. We utilized a combination of molecular, biochemical, and genetic methods to reveal a new biological function for SCUBE3 as a key regulator of fast muscle precursors. SCUBE3 potentially acts as a coreceptor at the plasma membrane to promote Fgf8 signaling for the differentiation of fast muscle precursors in zebrafish [9]

Despite the production of SCUBEs by endothelial cells [2, 5], ablation of any single *Scube* gene does not cause any overt vascular phenotype during development [20, 21, 45, 129]. Thus, SCUBEs may exhibit functional redundancy, or other *Scube* genes may compensate for a single gene knockout during embryogenesis. Owing to the fact that SCUBE proteins may interact as homodimeric or heterodimeric complexes [2, 5], it should be possible to infer whether SCUBEs cooperate genetically or functionally during vasculogenesis. We therefore initiated a study to dissect the mode of cooperation of *scube* genes in zebrafish [130]. Knockout alleles were generated by genome editing for each *scube* gene. As expected, no major phenotypes were observed in the vasculature of any single *scube* KO mutant, confirming the redundancy or compensatory effects of the *scube* genes in zebrafish development [130]. In contrast, the *scube1*/*scube2* double knockout shows severe defects in EC proliferation, filopodia extension and migration, which disrupt vascular lumen formation in vasculogenesis and angiogenesis, further impairing the development of distinct vasculatures in different organs [130]. These findings are in line with our recent studies showing that EC SCUBE2 acts as a coreceptor for VEGFR2 and regulates VEGF-induced tube formation and proliferation of ECs [23, 24]. In particular, SCUBE2 regulates VEGFR2-mediated signaling in the context of postnatal ischemia-induced angiogenesis [23] and also in hypoxic pathological tumor angiogenesis [24]. Further genetic, pharmacological and molecular studies in zebrafish suggest that Scube1 and Scube2 may collaborate at the level of cell-surface receptors to promote Vegfa signaling during embryonic vascularization (Fig. 6A) [130]. Additionally, our observations imply that it may be possible to treat VEGF-mediated proliferative vascular disorders by modifying VEGF signaling with therapeutic agents that target SCUBE1 and/or SCUBE2.

### Pathological functions

#### Breast cancer

**Fig. 7:**
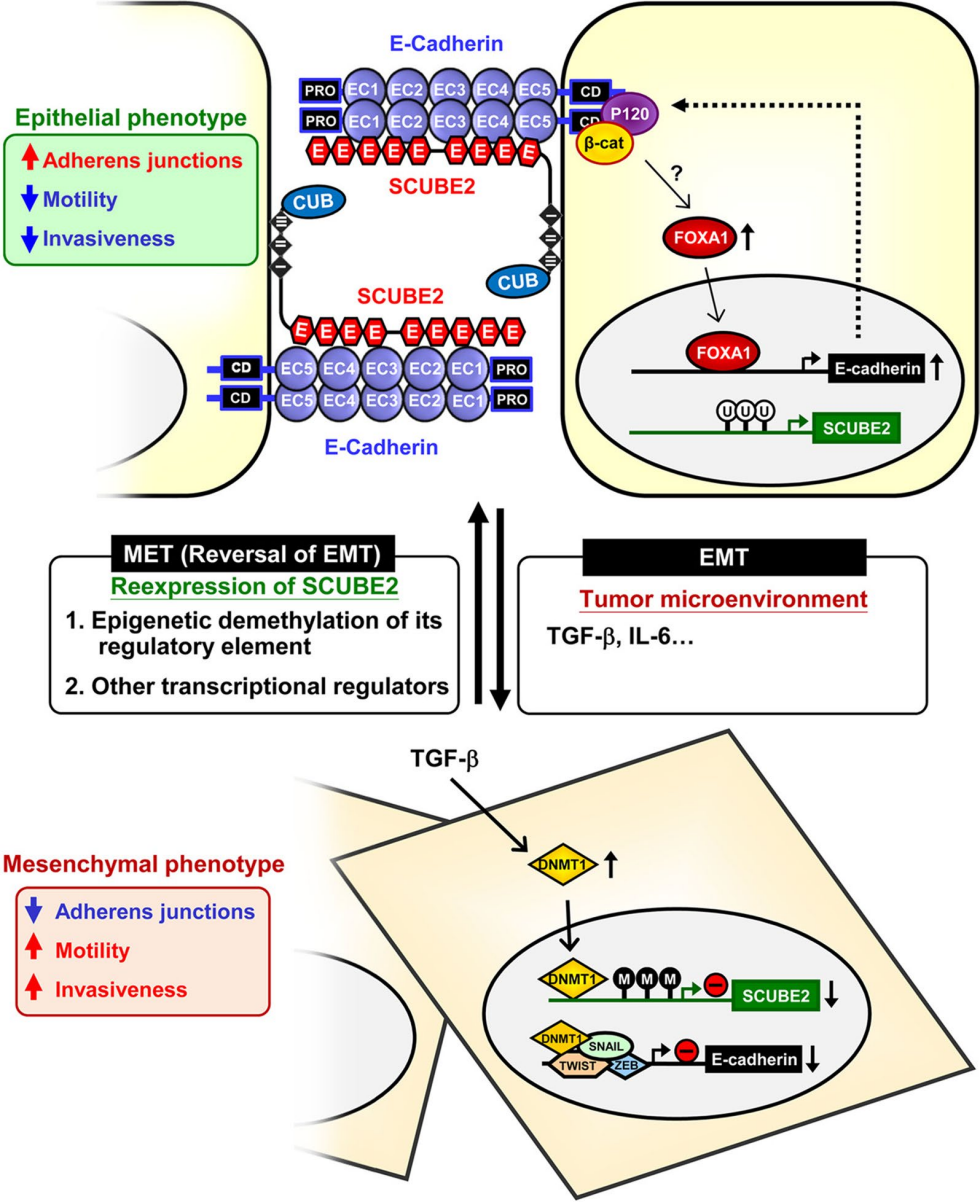
SCUBE2 functions as a tumor suppressor, preventing the migration and invasion of breast cancer cells by reversing epithelial-mesenchymal transition (EMT). By promoting the expression of forkhead box A1, an E-cadherin positive regulator, and subsequent transactivation of E-cadherin, SCUBE2 increases the formation of E-cadherin-containing adherens junctions. This process causes breast cancer cells to undergo epithelial transition by reversing EMT. During EMT, *SCUBE2* is epigenetically silenced by binding of DNA methyltransferase 1 at its CpG islands

The first clinical implication of *SCUBE2* in breast cancer biology was from a gene expression profiling study in which the authors made cross-platform comparisons of lists of genes [103, 104] or recurrence score models [105, 106]. The authors concluded that the breast cancer gene expression profiles overlap by only one gene, *SCUBE2* [102]. We discovered that endogenous SCUBE2 protein is expressed on the ductal epithelial or vascular EC surface of healthy breast tissue using immunohistochemistry with an anti-SCUBE2 polyclonal antibody. Additionally, in terms of disease-free survival, patients with positive SCUBE2-expressing tumors (55%; 86/156) have a superior prognosis to those with negative SCUBE2-expressing tumors. Our clinical association and functional studies also suggest that SCUBE2 may be a valuable biomarker indicating good clinical prognosis. Breast cancer cell proliferation and tumor growth were found to be significantly influenced by changes in SCUBE2 protein levels [112]. Molecular and biochemical analyses revealed that SCUBE2 protein is cleaved into N-terminal and C-terminal fragments by MMP-2 protease. The N-terminal EGF-like repeats mediate calcium-dependent cell–cell homophilic adhesion, interact with E-cadherin (a master tumor suppressor), and inhibit the β-catenin signaling pathway, whereas the C-terminal CUB domain directly bind to and antagonize BMP activity in an autocrine manner [22, 112]. During TGF-β-induced epithelial–mesenchymal transition (EMT), *SCUBE2* mRNA is downregulated in response to hypermethylation of CpG islands by DNA methyltransferase 1. By causing feed-forward induction of a positive regulator of E-cadherin, forkhead box A1, ectopic SCUBE2 overexpression can stabilize and facilitate E-cadherin-containing adherens junction formation. In turn, this action leads to epithelial transition and inhibits the migration and invasion of aggressive MDA-MB-231 breast cancer cells (Fig. 7; Table 4) [51].

**Table 4:** Roles of the SCUBE family in cancer biology

	Type	Suppressor/enhancer	Expression status	Signaling pathway	Type of studies	Summary	References
SCUBE1	BC	–	Upregulation	–	Clinical: D-dimer level, serum ELISA (normal; BC = 33; 50)	1. SCUBE1 level was higher in BC patients, especially the HER2^+^ group 2. SCUBE1 might be a biomarker of hypercoagulability in BC	[198]
	GC	–	Upregulation	–	Clinical: ELISA (normal: GC = 31; 52)	1. Plasma SCUBE1 was significantly increased in GC patients 2. SCUBE1 level did not differ whether metastasis occurred or not	[199]
	PC	Suppressor	–	NOTCH2	˙In vitro: proliferation ˙In vivo: xenograft mouse model, IHC	SCUBE1 OE in CAFs resulted in smaller tumor size and less invasion	[200]
		–	–	Hedgehog	In vitro: microarray, ELISA, qRT-PCR	SCUBE1 was upregulated after treatment with Smoothened agonist in primary prostate stromal cells	[201]
	SCLC	–	–	miR‐22‐3p/WRNIP1	In vitro: colony formation, apoptosis, proliferation, reporter assay	SCUBE1 was upregulated in miR-22-3p OE SCLC cells	[202]
	RCC	–	Upregulation	–	Clinical: plasma analysis (normal: RCC = 25; 23)	SCUBE1 level was significantly upregulated in RCC patients	[203]
SCUBE2	BC	Suppressor	Downregulation	BMP2	˙Clinical: IHC (n = 156), survival rate ˙In vitro: proliferation, IP, reporter assay ˙In vivo: xenograft mice model	1. Survival rate was reduced in SCUBE2-negative BC patients 2. SCUBE2 OE suppressed cancer cell proliferation and tumor growth 3. Although the C-terminal region of SCUBE2 interacted with BMP2 as an antagonist, the N-terminal was critical for extracellular secretion of SCUBE2	[112]
		Suppressor	High expression in epithelial breast cancer cells	1. E-cadherin 2. BMP2/Smad1/5/8	˙In vitro: domain analysis, cell growth, cell aggregation, IP, IF ․In vivo: xenograft mice model (n = 10 for SCUBE2-ty97 groups; n = 7 for SCUBE2-D4 groups)	1. SCUBE2 OE repressed cell growth or tumor proliferation 2. SCUBE2-D4, which includes Cys-rich domain, CUB domain and C-terminus, was responsible for antagonizing BMP signaling 3. EGF repeats of SCUBE2 were involved in calcium-dependent homophilic interaction and heterophilic interaction with E-cadherin	[22]
		Suppressor	Low expression in aggressive breast cancer cells	1. EMT 2. FOXA1/E-cadherin- β-catenin 2. DNA methylation	In vitro: migration, cell aggregation, reporter assay, IF, IP, ChIP, DNA methylation	1. SCUBE2 could enhance epithelial phenotypes, including upregulating E-cadherin expression and reducing migration ability 2. During TGF-β1 induced EMT, the CpG island of SCUBE2 promoter was methylated and thus was repressed	[51]
		Suppressor	Downregulation	DNA methylation	˙In vitro: viability, migration, DNMT activity ˙Clinical: IHC (tumor; adjacent = 10; 10), *SCUBE2* methylation	1. SCUBE2 was epigenetically modified by DNMT in breast cancer, thus downregulated 2. SCUBE2 KD repressed EMT characteristics 3. EGCG could rescue SCUBE2 loss by inhibiting DNMT expression and activity	[161]
		Suppressor	Downregulation	circ_SETD2/miR-155-5p	˙Clinical: qRT-PCR (tumor: adjacent = 54: 54) ˙In vitro: cell cycle, proliferation, migration, apoptosis, reporter assay ˙In vivo: xenograft mice model (control; circ_SETD2 OE = 15: 15)	1. SCUBE2 was downregulated in breast cancer cells 2. SCUBE2 OE resulted in cell cycle arrest, slow growth, enhanced apoptosis and impaired migration ability 3. Circ_SETD2 interacted with miR-155-5p, which targeted SCUBE2	[142]
		Suppressor	Downregulated in TNBC	C19MC miRNAs	˙Meta-analysis: miRNA-seq, RNA-seq, TCGA methylation array, GEO database, Oncomine database ˙In vitro: ChIP-seq	1. The expression of SCUBE2 was downregulated in TNBC and co-occurrence with NAT1 2. C19MC miRNAs could target SCUBE2 to contribute to triple-negative phenotype	[140, 141]
		Suppressor	Associated with ER^+^ or PR^+^ status	ER^+^ and PR^+^	˙Meta-analysis: TCGA, CCLE, and GEO database, CNV analysis ˙Clinical: IHC, qRT-PCR, survival rate	1. SCUBE2 was associated with ER^+^ or PR^+^ status 2. Deletion of SCUBE2 was associated with poor survival	[132–134, 138, 139, 204]
		Suppressor	Upregulated in non-metastatic groups	–	˙Meta-analysis: TCGA, METABRIC, and GEO database, IPA ˙Clinical: genomic microarray, gene expression microarray, qRT-PCR, FISH, IHC, RNA Chip, survival rate, mortality, recurrence	1. SCUBE2 was one of the overlapping genes among diverse multigene expression profiles for breast cancer 2. SCUBE2 was downregulated in metastasized tumors and significantly associated with poor survival rate 3. High expression of SCUBE2 was associated with reduced time to disease-related mortality 4. SCUBE2 was associated with immune-poor tumors	[131, 135, 205–210]
		–	–	SNP analysis	Clinical: cognitive performance, SNP analysis (normal; BC = 82; 138)	1. SCUBE2-related SNPs including rs1136966, rs4910440, and rs6486125 were investigated 2. SCUBE2rs6486125 was associated with attention, executive function, and mental flexibility	[211]
		–	–	hsa-miR-9-5p	˙Meta-analysis: miRNA-seq, RNA-seq, and clinical data from TCGA (normal adjacent tissue; BC tissue = 104; 1066) ˙In vitro: qRT-PCR	1. Hsa-miR-9-5p was upregulated in BC and could target SCUBE2 2. Patients with low SCUBE2 levels had a longer median survival time but much lower 10-year survival rate than those with higher levels	[137]
		Enhancer (in BCSC)	Upregulated in BCSC	NOTCH	˙In vitro: sphere formation assay, side-population, qRT-PCR, migration ˙In vivo: xenograft mice model (n = 6 each)	1. SCUBE2 was enriched in BCSC population and TNBC, and SCUBE2 OE enhanced cancer stemness, EMT and tumorigenesis 2. SCUBE2 could regulate cancer stemness via NOTCH signaling	[212]
	CRC	Suppressor	Downregulation	–	˙Clinical: qRT-PCR, WB, IHC, microarray (tumor; adjacent = 120; 120) ˙In vitro: proliferation, migration, apoptosis	1. SCUBE2 expression was reduced in CRC tissue and was associated with poor prognosis and survival rate 2. SCUBE2 OE led to inhibition of proliferation and migration	[146]
	EC	–	Downregulation	–	Clinical: qRT-PCR (n = 60)	SCUBE2 was decreased in grade-3 EC and was positively correlated with ERα, PR and PTEN levels	[150]
	GC	Suppressor	Downregulation	–	Clinical: qRT-PCR, WB, IHC, survival rate (tumor; adjacent = 124; 124)	1. SCUBE2 was significantly reduced in GC tissue 2. Decreased SCUBE2 was associated with poor prognosis and lower survival rate	[148]
	MG	Suppressor	Downregulation	SHH	˙Clinical: qRT-PCR, WB (normal; MG = 20; 20) ˙In vitro: cell viability, colony formation, migration ˙In vivo: xenograft mice model	1. SCUBE2 was downregulated in MG 2. SCUBE2 OE led to decreased cell viability, colony formation, migration and tumorigenesis 3. SCUBE2 OE inhibited SHH transduction	[145]
	NPC	–	Downregulation	–	Meta-analysis: EBI ArrayExpress	SCUBE2 was one of the hypermethylated and downregulated genes in NPC	[151]
	NSCLC	Suppressor	Downregulation	SHH	˙Clinical: qRT-PCR, WB (tumor; adjacent = 10; 10) ˙In vitro: apoptosis, migration	1. SCUBE2 was downregulated in NSCLC 2. SCUBE2 suppressed proliferation and migration, and enhanced apoptosis in NSCLC 3. SCUBE2 antagonized SHH signaling	[144]
	OSCC	Suppressor	Downregulation	–	˙Clinical: IHC, survival rate (n = 43) ˙Meta-analysis: GeneChip (n = 168), RNA-seq (n = 198)	1. Low level of SCUBE2 indicated poor prognosis 2. SCUBE2 tended to express in lymph node-negative tumors	[213]
	PTA	-	Downregulation	–	Meta-analysis: GEO (normal; PTA = 7; 34)	SCUBE2 was downregulated in PTA	[147]
	UBC	Suppressor	Downregulation	–	Clinical: IHC (n = 26), ISH	SCUBE2 was reduced in invasive regions and associated with lower survival rate	[149]
SCUBE3	BC	Enhancer	Upregulation	TGF-β1 TWIST1	˙Clinical: IHC, qRT-PCR, prognosis (tumor; adjacent = 30; 30) ˙In vitro: proliferation, migration ˙In vivo: xenograft mice model (control; KD SCUBE3 = 15; 15)	1. SCUBE3 was upregulated in breast cancer 2. SCUBE3 promoted EMT, migration and proliferation	[214]
		Enhancer	Upregulation	–	˙Clinical: IHC, survival rate (tumor; adjacent = 140; 140) ˙Meta-analysis: TCGA database (normal; tumor = 114; 1097)	1. SCUBE3 was expressed more in tumor tissue than adjacent tissue 2. Higher level of SCUBE3 was associated with lower survival rate regardless of BC subtypes	[177]
		Enhancer	Upregulation	–	Meta-analysis: TCGA (normal; HER^+^ BC = 20; 161)	SCUBE3 was identified as one of six genes in the risk scoring system for HER2^+^ BC	[215]
	ccRCC	–	–	–	˙Clinical: DNA methylation (n = 336), model validation (n = 64) ˙Meta-analysis: TCGA	SCUBE3 methylation was not found to be associated with ccRCC-specific survival	[216]
	HCC	Enhancer	Upregulation	TGFβ/PI3K/AKT/GSK3β	˙Meta-analysis: TCGA (normal; HCC = 114; 1097) ˙In vitro: proliferation, apoptosis, cell cycle, microarray, IP ˙In vivo: xenograft mice model (n = 10 each group)	1. SCUBE3 was upregulated in HCC, and *SCUBE3* KD led to inhibited proliferation, cell cycle arrest and apoptosis 2. SCUBE3 interacted with TβR-II and activated PI3K/AKT signaling pathway	[217]
	LC	Enhancer	Upregulation	MMP-2/MMP-9/TβR-II/Smad2/3	˙Clinical: qRT-PCR (tumor; adjacent = 18; 18), IHC (n = 60) ˙In vitro: IP, protein array, migration ˙In vivo: xenograft mice model (control; shSCUBE3 = 8; 7), tail vein assay of cancer metastasis (control; shSCUBE3 = 6; 6)	1. SCUBE3 was enriched in LC, and SCUBE3 KD led to decreased migration, tumorigenesis and metastasis 2. SCUBE3 bound to TβR-II via the CUB domain and induced downstream signaling 3. SCUBE3 CUB domain was cleaved by MMP-2 and MMP-9	[48]
		Enhancer	–	Angiogenesis	In vivo: xenograft mice model, MRI, IHC, RNA chip	1. SCUBE3 KD led to larger tumor size at week 1 and less vascular permeability at week 3 2. SCUBE3 KD caused less angiogenesis, EMT and CTCs	[218]
	MEL	Enhancer	Upregulation	SOX10/DEPDC1B	˙Clinical: IHC ˙In vitro: qRT-PCR, tube formation assay, ChIP, MS, microarray ˙In vivo: xenograft mice model (n = 5), lung metastasis assay (n = 7), metrigel plug assay (n = 4)	1. SCUBE3 was upregulated in MEL and acted as a pro-angiogenetic factor 2. DEPDC1B regulated ubiquitin ligase CDC16 to stabilize SCUBE3, thus facilitating SCUBE3 expression and secretion	[175]
	MG	Enhancer	Upregulation	AKT	˙Clinical: IHC (normal; MG = 3; 3) ˙In vitro: microarray, proliferation, apoptosis	1. SCUBE3 was downregulated in MG cells after treatment with asterosaponin CN-3 2. SCUBE3 KD led to cell cycle arrest in G0/G1 phase and inhibited proliferation	[179]
	NSCLC	Enhancer	Upregulation	–	Clinical: IHC, survival rate (tumor; adjacent = 119; 119)	SCUBE3 was highly expressed in NSCLC and associated with low survival rate	[174]
	OS	Promoter	Upregulation	–	˙Clinical: survival rate (n = 60) ˙In vitro: cell cycle, proliferation	1. SCUBE3 was highly expressed in OS cell lines, and *SCUBE3* KD led to cell cycle arrest in G0/G1 phase and proliferation inhibition 2. High SCUBE3 expression was associated with lower survival rate in OS	[181, 219]
		–	–	miR-590-5p	In vitro: lncRNA-seq, mRNA-seq	SCUBE3 was downregulated in miR-590-5p OE OS cell line	[180]
	RCC	Suppressor	Downregulation	DNA methylation	˙Clinical: MeDIP (normal; RCC = 3; 9), CoBRA (normal; RCC = 24; 60), qRT-PCR ˙In vitro: colony formation	1. Promoter of *SCUBE3* was highly methylated in RCC and was correlated with lower survival probability 2. SCUBE3 KD led to enhanced anchorage-independent growth potential	[183]
	SACC	–	Downregulation	miR-885-5p	˙Clinical: mRNA-seq, miRNA-seq, qRT-PCR (tumor; adjacent = 6; 6), IHC (n = 20) ˙In vitro: reporter assay	1. SCUBE3 was downregulated in SACC 2. The 3’UTR of SCUBE3 contained the miR-885-5p target site, and miR-885-5p was upregulated in SACC	
							[220]

*AKT* AKT serine/threonine kinase; *BC* breast cancer; *BCL2* B-cell leukemia/lymphoma 2; *BCSC* breast cancer stem cell; *BMP* bone morphogenic protein; *CAF* cancer associated fibroblast; *CCLE* Cancer Cell Line Encyclopedia; *ccRCC* clear cell renal cell carcinoma; *CEBPB* CCAAT/enhancer binding protein-β; *ChIP* chromatin immunoprecipitation; *C19MC* chromosome-19 micro-RNA cluster; *CoBRA* combined bisulfite restriction analysis; *CRC* colorectal carcinoma; *CTC* circulating tumor cell; *CUB* complement C1r/C1s, Uegf, Bmp1; *circ_SETD2* circular RNA circ_SETD2; *CNV* copy number variation; *DEPDC1B* DEP domain containing 1B; *DNMT* DNA methyltransferase; *EBI* European Bioinformatics Institute; *EC* endometrial cancer; *EGCG* (−)-epigallocatechin-3-gallate; *EGF* epidermal growth factor; *ELF5* E74 Like ETS transcription factor 5; *ELISA* enzyme-linked immunosorbent assay; *EMT* epithelial-mesenchymal transition; *ER* estrogen receptor; *FISH* fluorescence in situ hybridization; *FOXA1* forkhead box A1; *GC* gastric cancer; *GEO* Gene Expression Omnibus database; *GSK3β* glycogen synthase kinase-3 β; *HCC* hepatocellular carcinoma; *HER2* human epidermal growth factor receptor 2; *HR* hormone receptor; *IF* immunofluorescence; *IHC* immunohistochemistry; *IP* immunoprecipitation; *IPA* ingenuity pathway analysis; *ISH* in situ hybridization; *KD* knock down; *LC* lung cancer; *MeDIP* methylated DNA immunoprecipitation; *MEL* melanoma; *METABRIC* Molecular Taxonomy of Breast Cancer International Consortium; *MG* malignant glioblastoma; *MMP* matrix metalloproteinase; *MRI* magnetic resonance imaging; *MS* mass spectrometry; *NFIB* nuclear factor I B; *NPC* nasopharyngeal carcinoma; *NSCLC* non-small cell lung cancer; *OE* overexpression; *OS* osteosarcoma; *OSCC* oral squamous cell carcinoma; *PC* prostate cancer; *PGR* progesterone receptor; *PI3K* phosphoinositide 3-kinase; *PR* progesterone receptor; *PTEN* phosphatase and tensin homolog; *PTA* parathyroid adenoma; *qRT-PCR* quantitative reverse transcription-polymerase chain reaction; *RCC* renal cell carcinoma; *REST* RE1-silencing transcription factor; *SACC* salivary adenoid cystic carcinoma; *SCLC* small‐cell lung cancer; *SNP* single-nucleotide polymorphism; *SOX10* SRY-box transcription factor 10; *TβR-II* transforming growth factor-β (TGF-β) type II receptor; *TCGA* The Cancer Genome Atlas database; *TGF-β1* transforming growth factor beta 1; *TNBC* triple-negative breast cancer; *UBC* urothelial carcinoma of bladder; *UTR* untranslated region; *WB* Western blot; *WLS* Wnt ligand secretion mediator; *WRNIP1* WRN helicase interacting protein 1

In several clinical studies, SCUBE2 was found to be more highly expressed in non-metastatic tumor tissues than in normal tissues [131]. The expression of SCUBE2 predicts a favorable outcome in epidermal growth factor receptor (EGFR)-negative and estrogen receptor (ER)α-positive breast cancers [132–135]. Furthermore, low expression of SCUBE2 is associated with reduced time to disease-related mortality [135–139] and contributes to tumorigenesis in triple-negative breast cancer [140, 141]. The expression of SCUBE2 is also correlated with levels of microRNAs, such as hsa-miR-9-5p and miR-155-5p [137, 142]. In addition, SCUBE2 expression is upregulated in breast cancer stem cells, promotes EMT, and enhances metastasis of triple-negative breast cancer by promoting NOTCH signaling. [143]. These findings imply that SCUBE2 may be a potential druggable molecular target for treating triple-negative breast cancer that has spread and become aggressive (Table 4). Moreover, SCUBE2 expression and function in different types of breast cancer appear to be dynamically regulated at various stages of disease progression. In the earlier stages, SCUBE2 acts as a tumor suppressor, but is downregulated later during TGF-β-induced EMT. Most importantly, SCUBE2 is expressed in breast cancer stem cells and plays a role in the metastasis of triple-negative breast cancer. Since the roles of SCUBE2 in breast cancer appear to be complex and context-dependent, further investigations are needed on this topic.

#### Other cancer types

In several types of cancer, SCUBE2 is thought to act as a tumor suppressor. For instance, SCUBE2 is commonly downregulated in non-small-cell lung cancer (NSCLC) [144], glioma tissues [145], colorectal cancer tissues [146], parathyroid adenoma tissues [147], gastric carcinoma tissues [148], and invasive regions of bladder cancer [149]. Moreover, loss of SCUBE2 expression in different cancers is associated with pathological progression, in terms of clinical stage, tumor size, vascular invasion, lymph node status, depth of invasion and histological grade, and it is an independent predictor of poor prognosis [148–151]. Meanwhile, overexpression of SCUBE2 inhibits the proliferation of NSCLC, glioma, and colorectal cancer cells, and it also suppresses cell migration and invasion [144–146]. Kaposi sarcoma is a rare type of cancer that develops in the lining of blood and lymph vessels. A whole-genome sequencing and genome-wide linkage analysis identified two homozygous variants in a familial clustering of classical Kaposi sarcoma in an Iranian family. One identified variant is in *SCUBE2* (c.1489G > A [p.Pro497Ser]), and the other is in *CDHR5* [c.2269C > T (p.Gly757Ser)], which encodes a Mucin protein (Table 4) [152].

#### Tumor angiogenesis

Angiogenesis can be a double-edged sword in health and disease, as it is centrally involved in both normal tissue homeostasis and tumorigenesis, supporting the growth and dissemination of cancer cells. SCUBE2 acts as coreceptor for VEGFR2 expressed on the surface of ECs, which enhances VEGF-mediated signaling in adult angiogenesis [23]. However, it remains unclear whether SCUBE2 plays an important role in pathological angiogenesis. To address this issue, we performed a study investigating the pathological role of SCUBE2 in the vasculature of tumors. Our immunohistochemistry results showed that SCUBE2 is more strongly expressed in the ECs of several types of carcinomas compared to those in normal tissues. We also established tumor models using Lewis lung carcinoma cells (LLC), melanoma cells (B16F10), and mouse testis Leydig tumor cells (MLTC-1) that express very low levels of or no SCUBE2. These models showed that genetic knockout of SCUBE2 in ECs significantly decreases tumor vascular density and reduces xenograft tumor growth [143]. The abnormalities in angiogenesis seen in *Scube2*-knockout mice suggest that tumor cells should become starved of nutrients and oxygen, leading to apoptosis and necrosis. Correspondingly, *Scube2*-knockout mice display increased apoptosis (TUNEL assay) and markedly lower levels of tumor cell proliferation (Ki67 immunostaining) compared to wild types [143]. Together, these findings suggest that endothelial SCUBE2 is essential for angiogenesis that ensures tumor cell survival (Fig. 5).

#### Cardiovascular diseases

SCUBE2 is significantly downregulated in ECs after in vitro tumor necrosis factor-α and interleukin-1β treatments and after in vivo lipopolysaccharide injection [2]. Therefore, SCUBE2 may play important roles in inflammation-related diseases. The functions of SCUBE2 in inflammation-related diseases have been explored in several studies. In one study, a co-culture model of the blood–brain barrier was exposed to *Mycobacterium tuberculosis*, which damages the barrier and decreases the expression levels of tight-junction protein, type IV collagen, and SCUBE2. In addition, the SCUBE2-related HH pathway is downregulated by *M. tuberculosis* stimulation. The molecular mechanism of this phenomenon was also determined. Intriguingly, downregulation of SCUBE2 after exposure to *M. tuberculosis* limits release of SHH from astrocytes and its delivery to the protein patched homolog 1 (PTCH1) at the surface of ECs [153]. Another study showed that traumatic brain injury leads to the induction of MMP-9, which inhibits SCUBE2-mediated SHH release. These events cause damage to tight junctions and disrupt the blood–brain barrier [154]. In other work, researchers showed that in patients with dyslipidemia and type 2 diabetes, SCUBE2 is highly expressed and positively correlated with endothelin-1, a potent vasoconstrictor peptide produced by vascular ECs [155]. Moreover, SCUBE2 is upregulated in the injured carotid artery and aortic sinus after carotid artery ligation in wild-type mice and low-density lipoprotein receptor-knockout mice with high-fat-diet-induced atherosclerosis [156]. In a recent clinical study, the authors identified a missense variant (rs61751342) that co-segregates with carotid plaques. This variant is located in *DENND2B* and is an expression quantitative trait locus for SCUBE2 in the atrial appendage [115]. Despite the lack of detailed studies exploring the molecular mechanism of SCUBE2 in arteriosclerosis, it has been suggested that SCUBE2 may play an important role in the development of atherosclerotic plaques [115, 156].

Our previous studies showed that SCUBE2 is expressed in vascular ECs, where it genetically interacts with SCUBE1 and functions as a coreceptor for VEGFR2 to regulate VEGF-induced angiogenesis [23, 24, 130]. Furthermore, we showed that soluble SCUBE1 actively participates in thrombogenesis [20, 62, 72, 86]. Since thrombi frequently form in the atrial appendage of patients with atrial fibrillation or mitral valve disease [157], SCUBE2 may be considered as a potential therapeutic target for cardiac disease [115]. Nevertheless, functional studies are still needed to validate and further elucidate the putative roles of SCUBE2 in the pathological development of these heart conditions. Another genetic study revealed that Tibetans who live at high altitudes often carry an 662-bp intronic insertion in *SCUBE2* that is linked to improved lung performance [158]. This genomic structural variant was therefore postulated to contribute to high-altitude adaptation. Our studies showed that *SCUBE2* is upregulated by HIF-1α at both the mRNA and protein levels in lung ECs and that endothelial SCUBE2 acts as a novel coreceptor for VEGFR2 and potentiates VEGF-mediated signaling in adult angiogenesis (Fig. 5) [23]. Thus, our findings provide a likely mechanistic explanation for the observed association between the *SCUBE2* structural variant and better lung function in Tibetans [158].

#### Autoimmune disease

Multiple studies have linked SCUBE2 to different autoimmune diseases, including systemic sclerosis and rheumatoid arthritis. One recent study investigated the association of SCUBE levels with ultrasonographic skin thickness and other clinical characteristics in systemic sclerosis patients. The serum level of SCUBE2 was negatively correlated with the forced vital capacity value, and it was positively correlated with the Duruöz Hand Index and serum C4 level [159]. In another study, synovial tissue of rheumatoid arthritis rats showed a high level of SCUBE2 and low level of miR-543, which can target SCUBE2 and interact with a long non-coding RNA called plasmacytoma variant translocation 1. Furthermore, downregulation of plasmacytoma variant translocation 1 induces apoptosis of rheumatoid arthritis fibroblast-like synoviocytes via the miR-543–SCUBE2 axis [160].

### Translational application and therapeutic strategy

We recently demonstrated that EC-surface SCUBE2 is an essential VEGFR2 coreceptor in the context of pathological tumor angiogenesis [23]. Therefore, we further examined the possibility of pharmacologically treating solid tumors to inhibit SCUBE2 surface expression with a function-blocking mAb against SCUBE2 (SP.B1) developed by conventional hybridoma technology. The SP.B1 antibody recognizes only murine and human SCUBE2 in ECs and specifically recognizes the spacer region, which essential for SCUBE2 anchoring on the cell surface. Importantly, the mAb does not cross-react with SCUBE1 or SCUBE3. Treatment with SP.B1 in mice significantly decreased the growth and the vascular density of xenografted melanoma, as well as lung, pancreatic, testis, and colorectal tumors [143]. Further mechanistic investigations showed that SP.B1 binding to SCUBE2 causes the protein to be internalized for lysosomal degradation. This internalization reduces the SCUBE2 cell-surface level, attenuating VEGF binding and downstream signaling, as indicated by phosphorylation of VEGFR2 and stimulation of AKT/MAPK (Fig. 5). Another crucial finding in this study was that combination treatments of the anti-SCUBE2 mAb with chemotherapy (docetaxel and gemcitabine) or anti-VEGF antibody are more effective than single-agent therapies. Therefore, an anti-SCUBE2 mAb that induces protein internalization may provide a powerful adjuvant treatment to block tumor angiogenesis [143].

Global inactivation of SCUBE2 in mice does not appear to have any negative effects on the vascular system under normal physiological conditions. Since SCUBE2 is specifically upregulated by hypoxia and highly expressed in tumor vasculature, the anti-SCUBE2 mAb may provide a relatively nontoxic means of targeting tumor angiogenesis. As previously mentioned, we have shown that the mRNA and protein levels of SCUBE2, but not SCUBE1 or SCUBE3, are significantly upregulated after 12 h exposure of human umbilical vein endothelial cells (HUVECs) to hypoxia, and this upregulation occurs concomitantly with HIF-1α accumulation [23]. Additionally, our chromatin immunoprecipitation assay and promoter mutation analysis verified that endogenous HIF-1α can indeed bind to a DNA region containing a consensus HIF-binding motif (A/GCTGA) within the promoter region of SCUBE2, transactivating expression of SCUBE2 in HUVECs exposed to hypoxia [23]. Still, systematic toxicity studies are needed to determine the safety of combination treatments consisting of anti-SCUBE2 mAb and cytotoxic chemotherapeutic agents (e.g., gemcitabine or docetaxel) or anti-VEGF antibody (e.g., bevacizumab). Nevertheless, our studies to this point have shown that SCUBE2 acts a crucial VEGFR2 coreceptor during tumor angiogenesis, and targeting SCUBE2 on the cell surface may be an effective anti-angiogenic treatment strategy that may be especially useful in combination with cytotoxic chemotherapies.

Apart from its endothelial upregulation by pathological hypoxic conditions, our quantitative DNA methylation, methylation-specific PCR and chromatin immunoprecipitation analyses of breast tumor epithelial cells demonstrated that *SCUBE2* expression is inactivated due to DNA hypermethylation at CpG islands during TGF-β–induced EMT, and this hypermethylation is due to the recruitment and binding of DNA methyltransferase 1 [51]. This mechanism was also highlighted in a recent study on the regulation of *SCUBE2* methylation by (-)-epigallocatechin-3-gallate (EGCG), a highly abundant catechin in green tea. The authors found that treatment with EGCG reverses DNA methylation of *SCUBE2* in breast cancer [161]. In addition, the study showed that EGCG inhibits breast cancer cell viability and suppresses the migration and invasion capacities of the cells, while knockdown of endogenous *SCUBE2* blocks these inhibitory effects of EGCG in breast cancer cells. EGCG treatment was also shown to enhance *SCUBE2* gene expression, elevate E-cadherin expression and decrease vimentin expression. Furthermore, it was revealed that EGCG significantly decreases the DNA methylation status of *SCUBE2* by attenuating the expression and activity of DNA methyltransferase. These results suggest that pharmacological upregulation of the tumor suppressor *SCUBE2* might be a potential therapeutic approach for breast cancer.

## SCUBE3

### Regulation of gene expression and posttranslational control

SCUBE3 is the third and last member of the SCUBE protein family to be identified. By conducting a homology search, we identified and characterized *SCUBE3* in the human genome, as it shares an overall 60% protein identity and a similar protein domain organization with other SCUBE members (Fig. 1A, D). Quantitative RT-PCR and northern blot analyses revealed that the gene is prominently expressed in human primary cultured osteoblasts and mouse long bones, such as the humerus and femur; lower expression levels are present in human HUVECs and the heart [5]. In agreement with human *SCUBE3* expression in osteoblasts and long bones, mouse *Scube3* transcripts are expressed in the peripheral skeleton, including the ribs, limbs, and vertebra during cartilaginous condensation [129, 162, 163]. Similar to other SCUBE members, overexpressed human SCUBE3 protein exists as a secreted glycoprotein tethered to the cell surface that can form homodimers or heterodimers with other SCUBE members [5]. Secreted human SCUBE3 protein can be further proteolytically processed by an as yet unknown serum-associated protease to separate the N-terminal EGF-like repeats from the C-terminal CUB domain [5]. As seen with SCUBE1, SCUBE3 contains a minimal consensus sequence for furin-like protease (R_537_XXR_540_) within the spacer region, which might be responsible for its proteolytic cleavage in the presence of fetal bovine serum [5]. Based on its high expression in osteoblasts and long bones, we originally postulated that SCUBE3 might act locally and/or distally through a proteolytic mechanism to modulate bone cell homeostasis during development, repair and/or regeneration. In support of this hypothesis, recent human genetic studies have shown that bi-allelic inactivating variants in *SCUBE3* on chromosome 6p21.3 contribute to a developmental disorder involving, dental abnormalities, certain skeletal features, growth retardation, and a distinctive craniofacial appearance [21, 164]. Likewise, screening of mouse ENU-induced mutagenesis phenotypes led to the identification of recessive mutations in *Scube3* on chromosome 17qA3.3 that confer severe spine deformity, skeleton abnormalities, and altered bone metabolism (see below for details) [164, 165].

### Developmental and physiological function

#### Hair growth

According to whole-mount and radiolabeled in situ hybridization, mouse *Scube3* transcripts are primarily localized to the neuroectoderm at embryonic day (E)8.5. At E9.5 to E10.5, *Scube3* expression is observed in the branchial arches and frontonasal region, as well as the ventral dermomyotome of somites and limb buds [162, 163]. Of note, expression of *Scube3* in the central nervous system is restricted to ventral regions within the neural tube and cranial ganglia, including the trigeminal. In addition, mouse *Scube3* mRNA is expressed in a number of tissues during embryonic development, including ectodermal, endodermal and mesodermal derivatives (Table 3). *Scube3* transcripts are highly expressed in organs that develop via interactions between epithelium and mesenchyme, such as tooth germs and hair follicles [162, 163]. Indeed, a recent report showed that *Scube3* is expressed only in the growing but not resting hair follicles in dermal papillae [166]. In the study, new hair was induced in response to the injection of SCUBE3 protein, and pharmacological inhibition of TGF-β could rescue a mutant hair hyper-activating phenotype [166]. The authors therefore concluded that HH regulates mesenchymal niche function in the hair follicle via SCUBE3–TGF-β signaling [166]. Thus, SCUBE3 was shown to be a mesenchymal niche factor that activates hair growth.

Of note, the first targeted mutation allele of *Scube3* (*Scube3*^*tm1Dge/H*^) replaces the coding sequence of exons 2 and 3 with a *neomycin-lacZ* selectable marker cassette. Thus, only the EGF_1-2_ repeats are removed, and the allele still produces a partial Scube3 protein containing the C-terminal spacer region, CR and CUB domains. Since characterization of mice with this allele did not reveal any developmental abnormalities [163], it is possible that the partial Scube3 protein might retain functionality, at least during embryonic development [163]. Although this mutant line did not provide functional insights into the role of *Scube3* during mouse development, it is a useful reporter that has been used to verify the strong expression of *Scube3* in the epithelial compartments of developing teeth and hair follicles.

#### Chondrogenesis and osteogenesis

**Fig. 8:**
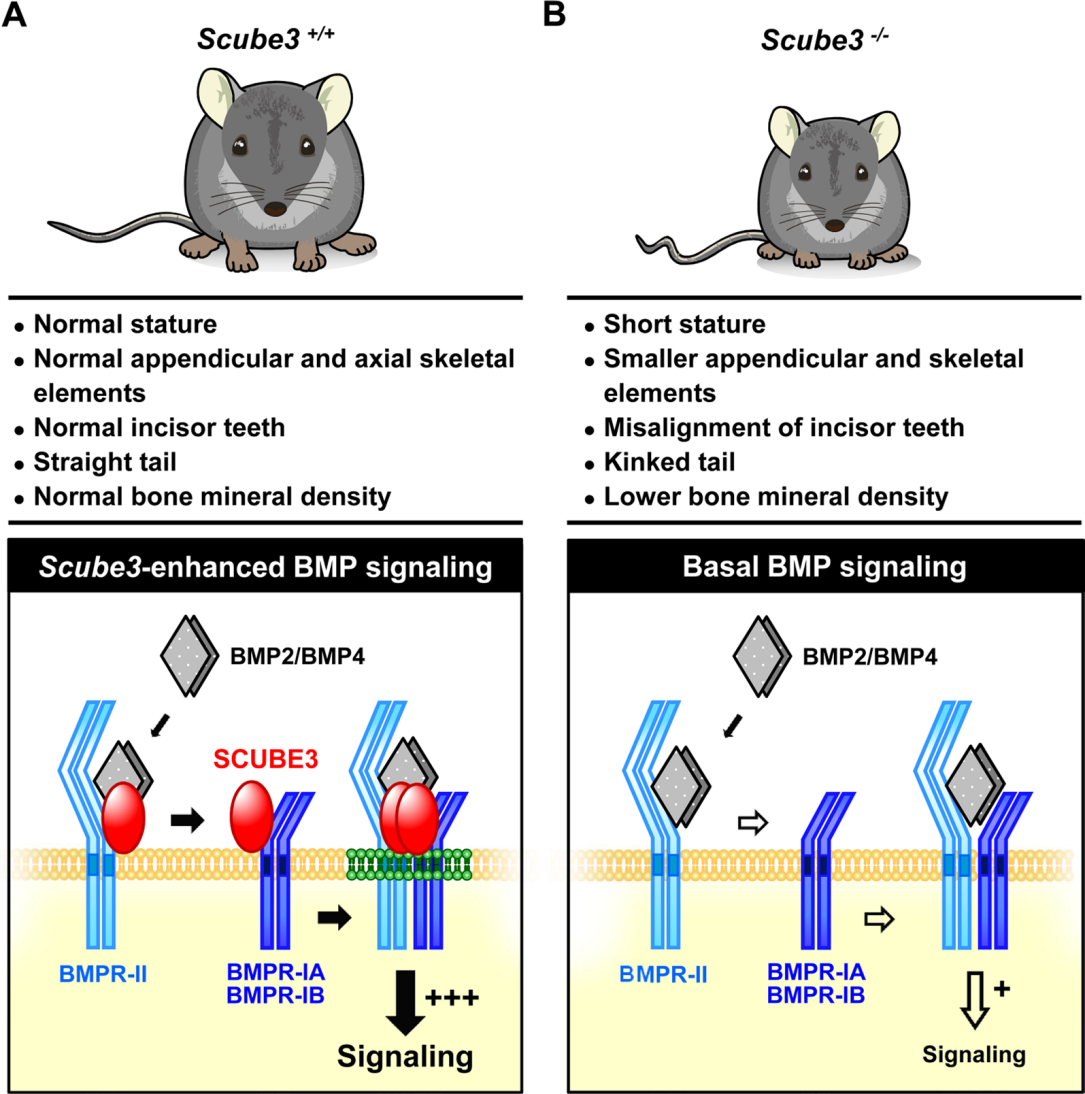
Schematic diagram depicts the role of SCUBE3 in enhancing the BMP signaling critical for endochondral bone formation. **A** When SCUBE3 (+/+) is present, it facilitates BMP2/4 ligand binding to type II receptors (BMPR-II) and recruits BMP type I receptors (BMPRIA or BMPRIB) to generate a SCUBE3-enhanced signaling complex. This complex relocates to the lipid raft (green-colored area) in response to BMP stimulation, which increases activation of the signaling cascade required for concerted endochondral bone formation. **B** In the absence of SCUBE3, lipid rafts show reduced BMP2 and BMP receptor levels. Thus, BMP downstream signaling and function are attenuated. *Scube3* deficiency in mice results in short stature, short-limbed skeletal dysplasia, misalignment of the front teeth, and kinked tail. In addition, bone mineral density is markedly lower in *Scube3-*deficient (−/−) mice compared to controls

**Fig. 9:**
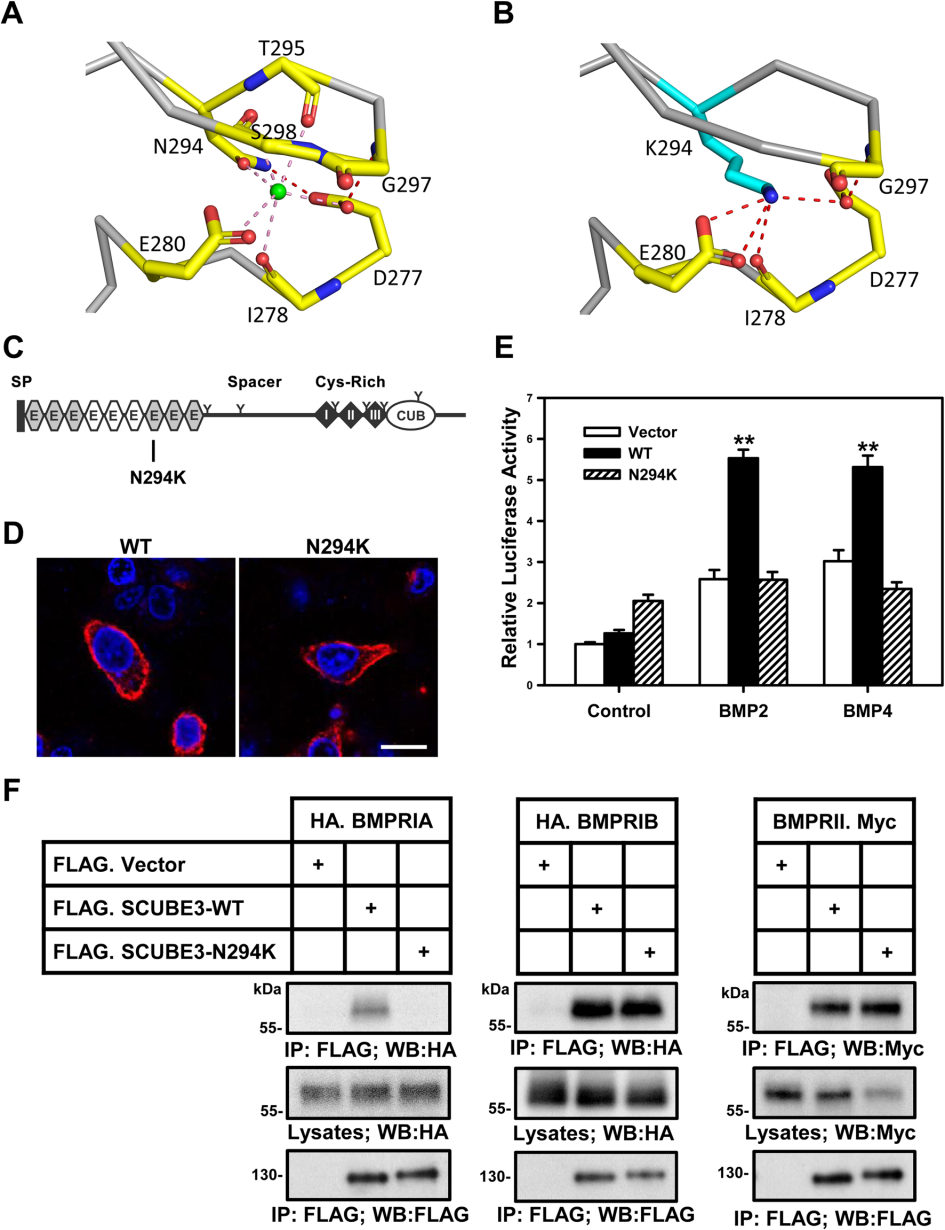
SCUBE3-N294K mutant has a defective interaction with the BMP type IA receptor, diminishing osteogenic BMP signaling. **A**–**B** Close-up of SCUBE3 cbEGF_7_ domains in N294 and N294K mutant. 3-D structure of mouse SCUBE3 seventh EGF-like domain (D277 to C316) was extracted from the AlphaFold2 model (AF-Q66PY1-F1-model_v3) [187]. Calcium ion (green sphere) was taken from the fifth EGF-like domain of human NOTCH1 (PDB 5FM9) [221] and coordinated by the oxygen atoms of D277, E280 and N294 side-chains and carboxyl atoms of I278, T295 and S298 backbones (shown as pink dotted lines with carbon atoms colored in yellow, oxygen atoms in red, and nitrogen atoms in blue). Additionally, hydrogen bonds (red dotted lines) form between the nitrogen atom of the N294 side-chain and the carboxyl atom of the D277 side chain and between the hydroxyl atom of D277 and the backbone nitrogen of G297 to stabilize the overall conformation of the cbEGF_7_ domain (**A**). For the N294K mutant, the lysine residue is shown in ‘stick-mode’ with carbon atoms colored in cyan. The mutant K294 forms a hydrogen-bond network (red dotted lines) with oxygen atoms from the side-chains of D277 and E280 and backbone of I278. Of note, the nitrogen of K294 possibly excludes and occupies the position of calcium. This figure was produced using PyMOL (Schrödinger, LLC. The PyMOL molecular graphics system, version 1.8, https://pymol.org) (**B**). **C** Protein domain structure depicting the amino acid exchange of N294K lying within the calcium-binding seventh EGF-like domain (cbEGF_7_) of SCUBE3. **D** Cell-surface distribution of SCUBE3-wild-type (WT) and -N294K protein. The expression plasmids encoding FLAG-tagged SCUBE3-WT or -N294K were transfected in HepG2 cells for 2 days. Immunofluorescence staining of cells with anti-FLAG (red) antibody. Nuclei were visualized by DAPI staining (blue). Scale bar = 10 μm. **E** SCUBE3-N294K mutation blunts BMP2/4-stimulated signaling activity. The BMP-responsive luciferase reporter (BRE-luc) and pRL-TK alone or in combination with the designated expression vectors were transfected into HepG2 cells. Luciferase activity was assessed 24 h after transgenic cells were treated with or without BMP2 or BMP4 (50 ng/ml). Firefly luciferase values were normalized to Renilla activity to show relative luciferase activity. The experiments were performed 3 times in triplicate. Data are mean ± SD. **p < 0.01. **F** Effect of the SCUBE3-N294K mutant on interactions with BMP signaling cascade receptors. In HEK-293T cells, HA-tagged BMPR-IA, HA-tagged BMPR-IB, or Myc-tagged BMPR-II constructs were co-transfected with plasmids expressing FLAG-tagged SCUBE3-WT or -N294K. In order to identify protein–protein interactions, transfected cell extracts were immunoprecipitated after 48 h and used for western blot analysis with the designated antibodies

To further investigate the developmental functions of SCUBE3, a second targeted *Scube3* allele was generated by replacing all of the coding exons with a ZEN-Ub1 promoter-driven expression-selection cassette [*lacZ-(pA)-hUbiPro-neo-(pA)*] [129]. The resulting *tm1(KOPM)Vlcg* allele contains a lacZ reporter for verification of its expression, and it was used to create heterozygous animals (*Scube3*^+/−^) on a C57/Bl6 background. Reporter activity domains in the *Scube3*^+/−^ animals were generally consistent with the embryonic expression domains reported previously for wild-type *Scube3* [162] or another *Scube3*^*tm1Dge/H*^ reporter [163]. Interestingly, mice that are homozygous for the *tm1(KOPM)Vlcg* allele (*Scube3*^*−/−*^) are born at expected Mendelian ratios, show no overt embryonic phenotypes, and are both viable and fertile [129]. Furthermore, mouse *Scube3* does not appear to affect the HH pathway, unlike zebrafish *scube3*, which cooperates with *Scube2* to modulate HH signaling [116]. The *Scube3*-knockout embryos retain normal HH signaling activity, as indicated by expression of *Ptch1*, a known downstream transcriptional target of the HH pathway. Therefore, mouse *Scube3*, at least on a C57/Bl6 genetic background, seems to be dispensable for gross embryonic development and survival. These results suggest functional redundancy of *Scube3* with other family members during development. However, the authors did not rule out a potentially exclusive role for the protein in postnatal tissue homeostasis or regeneration. In line with this suggestion, our analysis of this knockout mouse revealed that it has impaired BMP-mediated chondrogenesis and osteogenesis, which result in craniofacial and dental abnormalities, decreased body size, and faulty endochondral bone development (Fig. 8) [21]. Our molecular studies further demonstrated that membrane-associated SCUBE3 may function as a BMP2/4 coreceptor, attract BMP receptor complexes to raft microdomains, and enhance BMP signaling, perhaps by facilitating the interaction of BMP ligands with BMP type I receptors (Fig. 8) [21]. Consistent with these findings, mice homozygous for an ENU-induced mutant allele (*Scube3*^*N294K/N294K*^) exhibit skeletal abnormalities, including small body size, short kinked tails, abnormal digit positioning, malformations of the thoracic and lumbar vertebrae, and short femora, as well as other aberrations in parameters related to bone metabolism [165]. In this mutant allele, an asparagine-to-lysine exchange at protein position 294 (N294K) lies within the cbEGF_7_ repeat containing a calcium-binding consensus sequence [(D/N)-X-(D/N)-E/Q-X_*m*_- (D/N^*^)-X_*n*_-(Y/F) [17], where *m* or *n* are variable and the asterisk marks potential β-hydroxylation]. Our homology modeling suggested that the side-chain of β-hydroxylated N294 forms a hydrogen bond with D277 and serves as one metal-acceptor among oxygen atoms of other residues. In contrast, the N294K mutant forms a stronger hydrogen network with D277, I278 and E280, with the nitrogen of K294 likely occupying the position of calcium (Fig. 9A, B). Hence, the N294K exchange might lead to reduced calcium-binding capacity and/or conformational changes that affect the well-documented function of the cbEGF domains in mediating protein–protein interactions. Indeed, our molecular analysis showed that the SCUBE3-N294K mutant protein is expressed on the cell surface, but it does not readily form a complex with the BMP type IA receptor. This lack of interaction serves to abrogate osteogenic BMP2/4-induced signaling activity (Fig. 9C–F). Together, these results might provide a potential molecular explanation for the morphological abnormalities of the skeleton observed in this ENU-induced *Scube3*^*N294K/N294K*^ mutant [165]. Similarly, another ENU-induced mutant allele (*Scube3*^*C301Y*^) was reported to cause recessive spine deformities, such as severe kyphosis and kinked tail [164]. As with the N294K mutation, this C301Y mutation resides within the cbEGF_7_ domain, and it is expected to disrupt a key disulfide bond between conserved C2-C4 cysteines that is likely essential for proper domain folding and function. Consequently, this disease-causing SCUBE3 missense mutation impairs the interaction between SCUBE3 and SCUBE1 and reduces Smad signaling strength [164].

Together, the above mouse studies firmly implicate *Scube3* as a critical regulator in bone morphology and metabolism, as well as body size and weight. Likewise, a genome-wide association study in humans identified SNPs of *SCUBE3* among adult and pediatric height-associated loci [167, 168]. In pigs, *SCUBE3* is also associated with body height, body length, and rump circumference [169]. Of note, apart from bone-related phenotypes, *Scube3*^*N429K*^ mutant mice also showed disturbed renal function, hearing loss, altered inner and middle ear anatomy, and behavioral abnormalities [165].

#### Fast muscle development

In addition to performing studies on mice, we identified and characterized *scube3* in the zebrafish model system [9]. In that study, we found that *myod1* expression was selectively inhibited in lateral fast-muscle precursors upon *scube3* knockdown by antisense morpholino oligonucleotides, but it was largely unaltered in adaxial slow-muscle precursors. Immunofluorescence labeling of fast- but not slow-muscle myosin was also significantly reduced in *scube3* morphants. Further experiments revealed *fgf8* as a crucial modulator of zebrafish *scube3*-mediated rapid muscle development. Additional biochemical and molecular research then showed that SCUBE3 enhances FGF8 signaling by acting as a FGF coreceptor [9]. Thus, zebrafish Scube3 may modify Fgf8 signaling during development in order to serve as an important upstream regulator of fast fiber myogenesis (Fig. 6B).

### Pathological function

#### Cardiac hypertrophy

In addition to our loss-of-function studies in mice, we generated the first transgenic mice overexpressing SCUBE3 to further explore the function of SCUBE3 in vivo [170]. Expression was controlled by a type I collagen promoter, which induces strong expression of green fluorescent protein in isolated tail tendons and bone, but has relatively lower expression in other type I collagen-producing tissues [171, 172]. Male transgenic mice that overexpress SCUBE3 appear to grow normally from the embryonic period to adulthood. However, when the transgenic mice reach an age of ~ 8 months, the animals begin to display cardiac hypertrophy, as revealed by echocardiography and histopathological examination. Furthermore, pressure overload augments left-ventricle hypertrophy (i.e., it occurs more rapidly and severely) in transgenic animals. Our research indicates that the accelerated onset and progression of cardiac hypertrophy in transgenic mice may be due to pathological induction of SCUBE3 expression and elevated TGF-β1 level after pressure overload. Additionally, biochemical and molecular experiments showed that the C-terminal CR and CUB domains of SCUBE3 can interact with TGF-β1 and facilitate TGF-β1-mediated transcriptional activation [170]. Overall, this study suggested that upregulation of SCUBE3 modulates pathological TGF-β signaling to negatively impact cardiac growth and/or remodeling [170].

#### Lung cancer

In line with the pathological role of the SCUBE3-TGF-β signaling axis, our collaborative studies showed that soluble SCUBE3 can act via TGF-β to stimulate negative outcomes in lung cancer cells. In this context, SCUBE3 is cleaved by MMP-2 or MMP-9 to release its C-terminal CR and CUB domains. This fragment binds TGF-β receptors and elicits TGF-β-Smad2/3 signaling pathways, which drive EMT, migration and invasion of lung cancer cells as well as extracellular matrix deposition and angiogenesis [48]. Consistent with these findings, knockdown of SCUBE3 in a cellular model of NSCLC markedly reduces intratumor vessel density at the core region [173]. Moreover, microarray analysis of SCUBE3-knockdown lung tumors showed downregulation of angiogenic and EMT-related genes, including *MMP-2*, *MMP-9*, *MMP-14*, fibronectin, early growth response protein 1, hairy/enhancer-of-split related with YRPW motif protein 1, and interleukin 8 [173]. In agreement with these laboratory studies, a clinical association study correlated high SCUBE3 expression with low expression of the epithelial marker E-cadherin and high expression of the mesenchymal marker vimentin [174]. Importantly, the clinical data also showed that patients with high expression of SCUBE3 have a relatively poor survival rate, and SCUBE3 expression can serve as an independent prognostic factor for NSCLC [174] (Table 4).

#### Other cancer types

Consistent with the role of SCUBE3 in promoting angiogenesis in vitro and in vivo, a recent molecular study showed that DEP domain-containing 1B enhances melanoma angiogenesis and metastasis by stabilizing SCUBE3. DEP domain-containing 1B binds competitively with ubiquitin ligase cell division cycle 16, thereby preventing degradation of SCUBE3 via the ubiquitin–proteasome pathway [175]. Likewise, SCUBE3 promotes breast cancer progression via the activation of TGF-β1/TWIST1 signaling [176], and its expression can also independently serve as an indicator of poor prognosis in breast cancer [177]. In addition, SCUBE3 promotes hepatocellular carcinoma tumorigenesis by regulating cyclin E1 via the TGF-β/PI3K/AKT/GSK3β pathway [178]. SCUBE3 was also found to regulate glioma cell proliferation [179] and promote proliferation of osteosarcoma cells, and its expression is correlated prognosis of patients with osteosarcoma [180–182]. A public database search (www.proteinatlas.org) showed that high SCUBE3 protein expression can serve as a marker of unfavorable prognosis for human thyroid cancer (Table 4).

Besides these tumor-promoting functions of SCUBE3, genome-wide methylation analysis revealed that *SCUBE3* is a tumor suppressor that may be epigenetically inactivated in renal cell carcinoma [183]. In this study, the authors used a genome-wide analysis to identify genes frequently methylated and silenced in renal cell carcinoma. Using methylated DNA immunoprecipitation and whole-genome arrays combined with high-density expression arrays, the study showed that the promoter region of *SCUBE3* is frequently methylated in primary renal cell carcinoma tumor samples (19% of examined cases) [183]. Moreover, knockdown of *SCUBE3* increased the occurrence of anchorage-independent colonies (> 200 μm) in HEK-293 cells by 71%. Also, methylation of *SCUBE3* is associated with significantly increased overall risk of death (*P* = 0.009) and cancer death or relapse (*p* = 0.0046) in patients with renal cell carcinoma [183] (Table 4).

#### Skeletal disorders

**Fig. 10:**
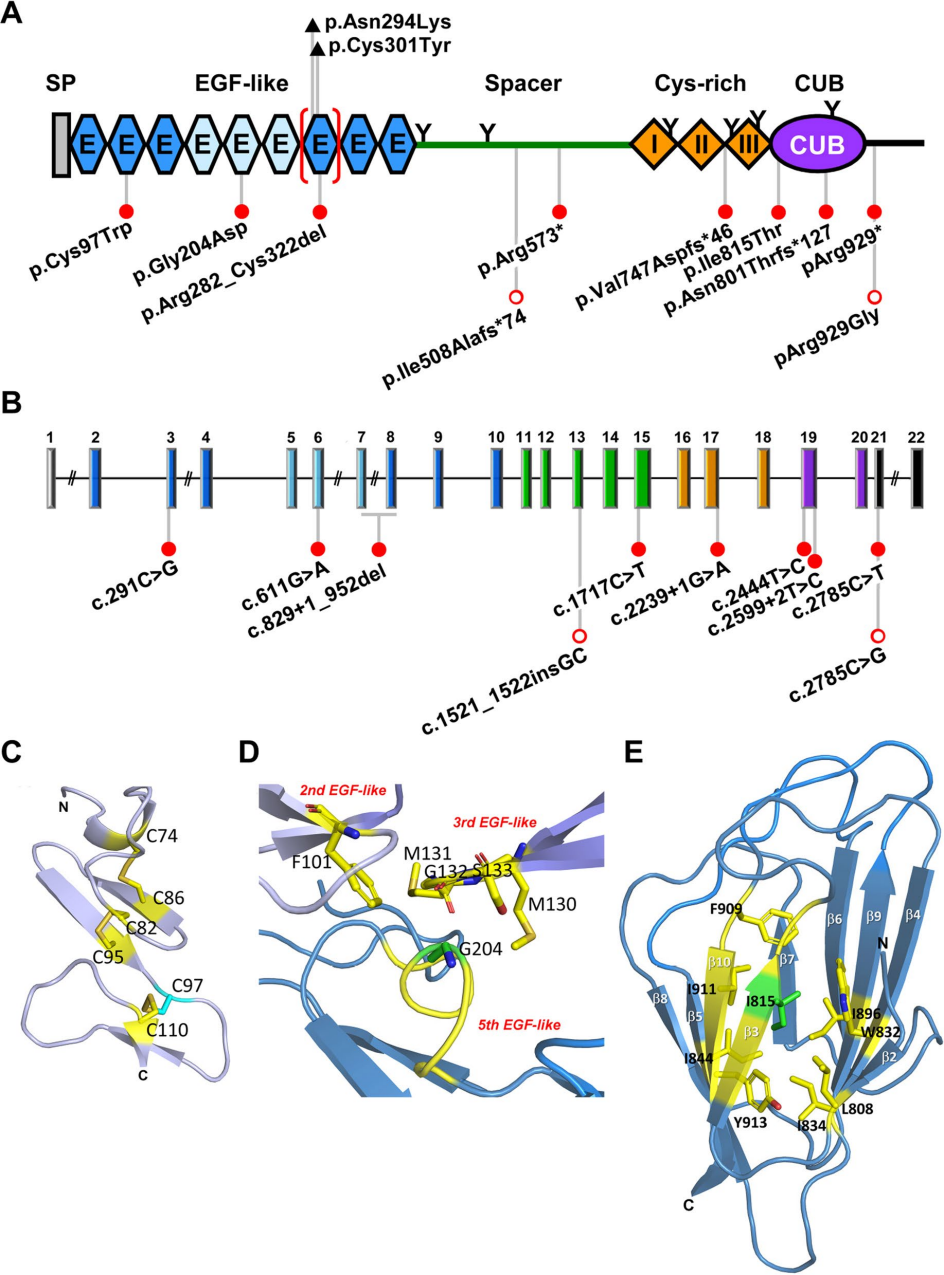
Genomic organization, protein domains, and predicted protein domain structure of SCUBE3. **A**–**B** Graphic display of the genomic organization of *SCUBE3* and the protein domain structure of its encoded product. Impacts of the disease-causing variants on protein coding are indicated. Our team first reported and characterized eight bi-allelic inactivating variants in *SCUBE3* from 18 affected individuals of nine unrelated families who showed a similar phenotype of distinctive craniofacial appearance, dental anomalies, skeletal features, and reduced growth (closed red circles) [21]. Another team then identified recessive mutations of *SCUBE3* from a patient with undiagnosed skeletal disease (open red circles) [164]. Two ENU-induced mutant alleles in *Scube3* associated with spine deformity including severe kyphosis and kinked tail [164], as well as skeletal abnormalities and altered bone metabolism [165] are also shown (closed black triangles above the diagram) (**A**). Genomic location of the disease-causing variants of *SCUBE3*. C.2599 + 2T > C is predicted to result in multiple transcripts with abnormal processing, with p.Asn801Thrfs*127 representing the most common out-of-frame product [21]. Each exon is shown with size to scale, but introns are not. Also, the colors were used to coordinate specific protein domains and their coding exons. For instance, the signal peptide sequence and corresponding exon 1 are both shown in grey. In addition, exons 19 and 20, and the encoded the CUB domain are shown in purple (**B**). **C–D** Residues C97 and G204 of human SCUBE3 from AlphaFold2 model. Three disulfide bonds between six cysteine side chains are shown in ‘stick-mode’ in the second EGF-like domain of human SCUBE3; for instance, C97 (in cyan) connects with C110. These six cysteine residues are crucial for disulfide connectivity in structure folding and stability (**C**). G204 (in green) located on the fifth EGF-like domain is in contact with F101 of the second EGF-like domain and residues M130, M131, G132 and S133 of the third EGF-like domain (atoms colored with carbon in yellow, oxygen in red and nitrogen in blue) in this model. The substitution of residue G204 into D204 may perturb the interactions between the three EGF-like modules in human SCUBE3 (**D**). **E** The position of residue I815 in the CUB domain of human SCUBE3. Ribbon model represents human SCUBE3 CUB domain (C804 to Y916) from AlphaFold2 prediction (AF-Q8IX30-F1-model_v3) [187] with the β-strands numbered (in white) based on the bovine spermadhesin CUB domain [224]. I815 is shown in green, and residues within a distance of 5 Å are in yellow. Side chains of these residues buried in the hydrophobic core are shown in ‘stick-mode’ with oxygen atoms in red and nitrogen atoms in blue. Of note, I815 and surrounding residues, including I896, W832, L808, I834, Y913, I844, I911 and F909, form a cluster of buried hydrophobic amino acids in the core of the domain. Replacement of I815 with a polar threonine residue is predicted to destabilize the structure of the CUB domain

In addition to the associations of aberrant *SCUBE3* expression with pathogenesis and clinical outcomes of various types of cancers, mutations in *SCUBE3* (GenBank: NM_152753, see Table 1) are associated with human skeletal disease. Eight bi-allelic inactivating variants in SCUBE3 were first discovered and characterized by our research in 18 affected people from nine independent families who shared the same phenotype of unusual craniofacial features, short stature, skeletal deformities and dental abnormalities [21]. Subsequently, another team identified different recessive *SCUBE3* mutations in a patient with undiagnosed skeletal disease [164] (Fig. 10A, B). The discovered variations, which comprised missense, frameshift, nonsense, and canonical splice site modifications as well as a complicated intragenic rearrangement, were dispersed throughout the entire coding sequence of SCUBE3. Two variants are expected to alter transcript processing [c.2239 + 1G > A (p.Val747Aspfs*46); c.2599 + 2T > C], and the other three are expected to cause truncations [c1717C > T (p.Arg573*); c.2785C > T (p.Arg929*); c.1521_1522insGC (p.Ile508Alafs*74)]. Total RNA isolated from the affected patient carrying c.2239 + 1G > A confirmed that a cryptic donor site of exon 17 was activated, four intronic flanking nucleotides were retention, and the coding sequence (p.Val747Aspfs*46) was prematurely terminated. However, a biological sample from the proband carrying the c.2599 + 2T > C variant was unavailable for functional validation. In order to confirm the aberrant splicing of the genomic region of SCUBE3 containing exons 18 to 21, we employed a minigene splicing assay for a number of mRNA variants expected to yield prematurely truncated proteins (p.Asn801Thrfs*127 was the most common out-of-frame product) [21] (Fig. 10A, B).

Four different missense changes were identified, all of which affect residues that are well-conserved among SCUBE members from different species [21]. A substitution c.291C > G (p.Cys97Trp) within the second EGF-like repeat affects one of the six conserved cysteines that form the disulfide bonds essential for stabilizing the EGF module structure. Thus, the protein should lose one of the three conserved disulfide bridges, causing a structural rearrangement of the EGF motif (Fig. 10C). Pathogenic variations in EGF-like modules at equivalent positions of fibrillin-1, fibrillin-2, and latent TGF-β binding protein 3 were previously reported [184–186], further supporting the putative functional importance of this variant. The second missense mutation [c.611G > A (p.Gly204Asp)] results in the exchange of a conserved glycine residue within the EGF_5_ module. The non-conservative substitution with aspartic acid is predicted to cause local structural conflicts with adjacent EGF-like modules (Fig. 10D). Based on the AlphaFold model of human SCUBE3 (CUB domains share 80% sequence identity) [187], a non-polar Ile^815^ reside is conserved and located on the β3 strand buried in the core of the CUB domain (Fig. 10E). The third missense variant [c.2444T > C (p.Ile815Thr)] involves a replacement of a non-polar isoleucine with a polar threonine. The structure of the hydrophobic core is expected to become unstable as a result of this replacement, which includes residues Leu^808^, Trp^832^, Ile^834^, Ile^844^, Ile^896^, Phe^909^, Ile^911^, and Tyr^913^ (Fig. 10E), thus perturbing the overall stability and folding of the CUB domain and its interaction with binding partners. The fourth missense mutation [c.2785C > G (pArg929Gly)] similarly changes a conserved positively charged arginine to a small glycine that might disrupt folding of the region and impair the proper function and binding ability of the CUB domain.

Only the cbEGF_7_ module is removed as a consequence of the complete deletion of exon 8 [c829_952del (p.Arg282_Cys322del)] by a complicated intragenic rearrangement in SCUBE3 (combined deletion and inverted duplication). Of note, two ENU-induced mutant alleles, *Scube3*^*N294K*^ or *Scube3*^*C301Y*^, with skeletal phenotypes might also have detrimental effects on the folding and function of the cbEGF_7_ module [164, 165]. Moreover, comparable intragenic structural rearrangements that lead to deletion of a single EGF-like module in fibrillin 1 (FBN1) or fibrillin 2 (FBN2) are known to significantly affect protein functions [188, 189].

We [21] and others [164] used transient transfection to further investigate the effects of a representative panel of variants affecting different domains of SCUBE3 (pCys97Trp, p.Arg573*, pIle815Thr, or pCys301Tyr) in terms of protein stability, secretion, membrane anchoring and function. These studies revealed four major findings. (1) The p.Cys97Trp variant protein shows less-efficient dimerization with itself and with wild-type SCUBE3 [21]. (2) The pArg573* truncated variant protein is neither expressed on the cell surface nor is it secreted in the medium, which suggests the protein might be mistargeted due to a trafficking defect and endoplasmic reticulum retention [21]. (3) The pIle815Thr mutant is defective as a BMP coreceptor and cannot promote BMP signaling in vitro [21]. (4) The pCys301Tyr variant has reduced interaction with SCUBE1 (i.e., deficient formation of the SCUBE1-SCUBE3 heterodimer) and fails to activate Smad signaling [164]. Together, these findings suggest that variants affecting the EGF-like or CUB domain can act through diverse mechanisms to cause functional deficiencies. Since mouse ENU mutagenesis screening and human genetic studies reproducibly and repeatedly show that disruption of the cbEGF_7_ module [due to destabilized domain folding by two disease-associated missense mutations (N294K or C301Y) or complete domain deletion by a genomic structural variant] causes functional deficits, one may conclude that the cbEGF_7_ module plays a critical, potentially calcium-dependent role in regulating SCUBE3 signaling and activity. Nevertheless, this hypothesis warrants testing in further in-depth structural and functional investigations.

Consistent with the common clinical features of individuals carrying bi-allelic loss-of-function *SCUBE3* variants, *Scube3* knockout animals show reduced growth associated with small forelimbs and hindlimbs. Furthermore, *Scube3* knockout mice typically exhibit misalignment of both upper and lower incisors, and the animals suffer from several craniofacial anomalies, such as a small forehead, short and narrow face, and short frontonasal and mandibular regions (Fig. 8) [21]. On occasion, it is also possible to find a knockout mouse with a hunchback spine or protruding ribs. Overall, these phenotypes recapitulate major features of *SCUBE3*-associated human skeletal disorders. Therefore, these clinical studies confirm *SCUBE3* as the first SCUBE member involved in Mendelian disorders. Our studies and those from another team positively identified these mutant *SCUBE3* alleles, which are primarily inactivating variants, as causing identifiable developmental abnormalities. Furthermore, our molecular characterization studies attribute this novel condition to the disruption of endochondral bone formation caused by defects in BMP signaling [21, 164].

### Translational application and therapeutic strategy

To date, aberrant upregulation of *SCUBE3* expression has been associated with unfavorable outcomes in various tumors, including NSCLC [181], breast cancer [181], metastatic melanoma [175], and osteosarcoma [181]. In contrast to these apparent oncogenic functions, *SCUBE3* might also serve as a tumor suppressor in the context of renal cell carcinoma. In patients with this disease, promoter methylation (epigenetically silenced) of *SCUBE3* is associated with increased risk of mortality or relapse [181]. Given that SCUBE3 is a secreted protein, it would be of great interest to determine whether elevated circulating soluble SCUBE3 protein might be associated with disease severity or progression. If so, the circulating protein may serve as a potential novel biomarker and/or a therapeutic target.

A recent study highlighted an interesting means of targeting SCUBE3 as a therapeutic strategy. In this study, SCUBE3 level was reduced by a new marine-isolated asterosaponin, which led to G1/S cell cycle arrest of glioma cells [179]. The authors showed that the newly identified asterosaponin, CN-3 (isolated from the starfish *Crocodylus novaeguineae*), could decrease the proliferation of two different glioma cell lines. Cellular and molecular studies revealed that the reduction of SCUBE3 expression by CN-3 is a key mediator of its anti-proliferative effects and cell cycle arrest via the AKT/p53/p21/p27/E2F1 signaling pathway [179]. Nevertheless, the function of SCUBE3 should be further explored in glioma, both in vitro and in vivo. In addition to chemical compounds that can modulate the expression of SCUBE3 under different pathological conditions, anti-SCUBE3 antibodies might be developed to specifically downregulate its cell-surface expression. The ultimate goal of any such method of targeting SCUBE3 would most likely be to suppress the pathological SCUBE3/TGF-β/Smad signaling pathway.

## Conclusions

**Fig. 11:**
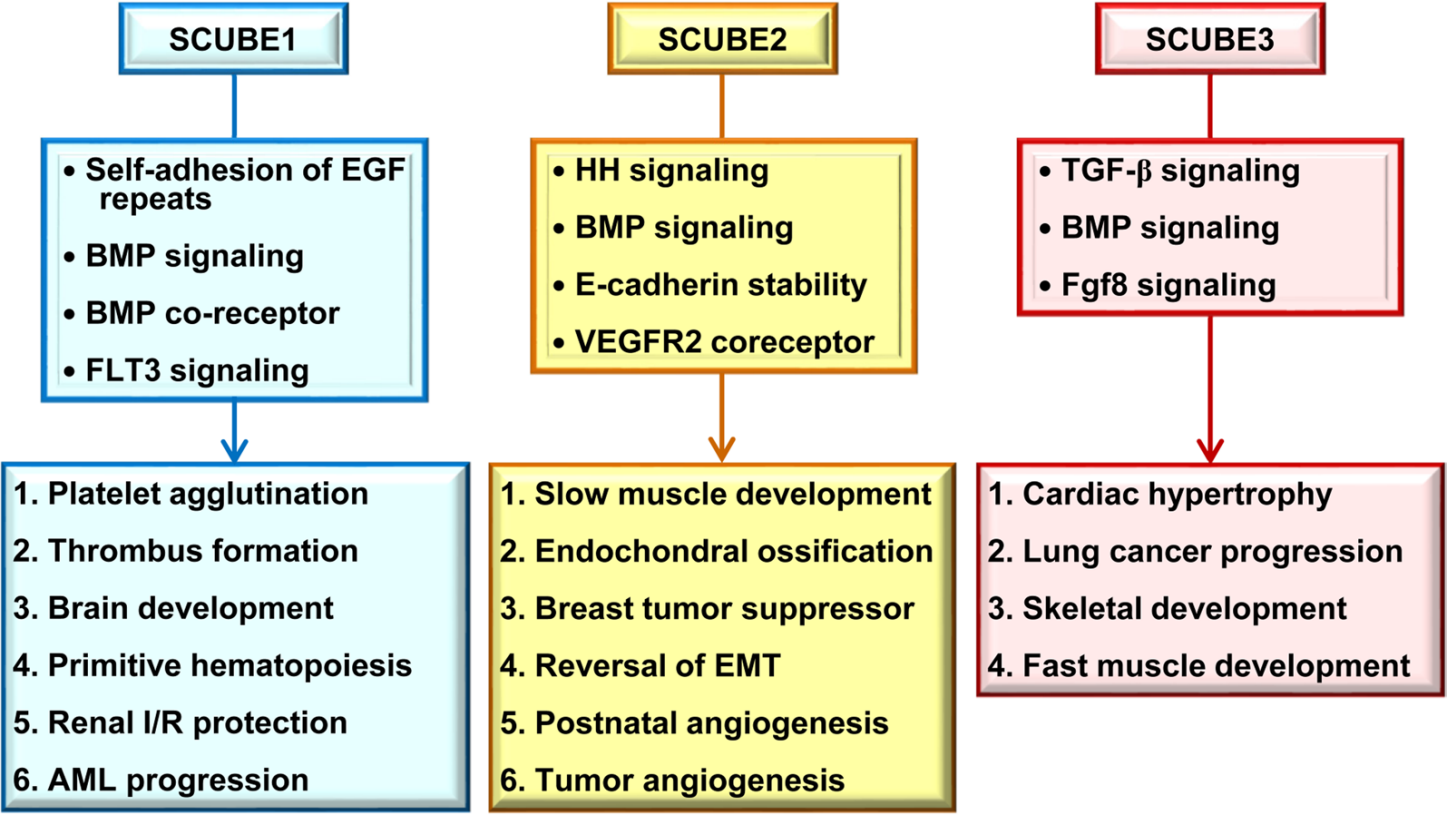
Summary of SCUBE-regulated growth factor signaling pathways and their involvements in physiological and pathological processes derived mainly from our studies. To date, different SCUBE members have been shown to modulate a diverse set of signal pathways activated by growth factors including BMP, FGF, TGF-β, VEGF, FLT3 ligand, and HH ligands. By these actions, SCUBE proteins participate in a wide variety of homeostatic and pathological processes

After two decades of research on the SCUBE family, many basic and translational discoveries have been made and reported (Fig. 11), especially regarding the roles of SCUBE proteins in various cancers (Table 4). However, many more biological functions of SCUBE proteins remain to be explored and will require further investigation. For example, the publicly available Allen Brain Atlas (https://portal.brain-map.org/) shows that SCUBE genes are expressed in various regions of the brain as well as spinal cord in mice and humans. Yet, little is known about the potential physiological or pathological functions of SCUBE proteins in neural tissues. This topic certainly warrants further in-depth neurobiological studies.

A Ramachandran plot analysis was performed to validate the accuracy of the SCUBE protein structures predicted by AlphaFold [190]; however, we still lack experimentally determined high-resolution structures of full proteins or key domains of SCUBE proteins. For instance, the impact of calcium binding on the structure and function of the cbEGF module or the cbCUB domain in SCUBE proteins is still undefined and uninvestigated. Furthermore, because SCUBE proteins interact with molecules in many different signaling pathways, we must unravel the structural basis of these interactions by conventional crystallization or by integrative approaches with cryogenic electron microscopy [191]. The structural insights derived from studies on SCUBE proteins in complex with signaling ligand binding partners (e.g., TGF-β, SHH, VEGF or FLT3 ligand) and their receptors might assist in the development of small-molecule inhibitors or antibody-based biologics. Such tools can potentially be used to antagonize disease-promoting signaling under pathological conditions, such as solid tumors or AML.

SCUBE1 is a multifunctional protein that has different functions based on its expression in different cell types. In several ischemic contexts, SCUBE1 is released into plasma and may be useful as biomarker for cardiovascular diseases [71, 81–83]. Its expression is also found in renal cancer, breast cancer and leukemia. Therefore, it could also be used as a biomarker in these cancers. However, detailed clinical analyses are needed to further validate the utility of circulating SCUBE1 levels as a biomarker for different conditions. In addition, although numerous studies have suggested that elevated plasma SCUBE1 levels might serve as biomarkers for different pathological conditions, these findings must be validated in prospective clinical trials on large patient cohorts.

SCUBE1 is also considered to be a promising therapeutic target in platelet activation and AML. With regard to platelet activation, an anti-SCUBE1 antibody was able to inhibit thrombus formation [72]. However, further studies are needed to uncover other promising methods to inhibit SCUBE1, such as small-molecule inhibitors to prevent thrombus formation. Likewise, in AML, anti-SCUBE1 ADC shows promising pre-clinical results in inhibiting disease progression [44]. Moreover, SCUBE1 could be an important target in AML and other types of leukemia due to its regulation by HOXA9, which is overexpressed in a broad range of leukemias [192]. Further analysis is needed to identify all the types of leukemia in which SCUBE1 is aberrantly upregulated. Furthermore, because SCUBE1 is expressed on the cell surface of leukemic blasts, methods such as chimeric antigen receptor T-cell therapy could be developed in addition to the ADC strategy that is currently being evaluated.

Another relatively unexplored field is the pathological implications and therapeutic targeting of the well-documented SCUBE2-mediated enhancement of HH signaling [113, 193]. Given that HH signaling plays a critical role in maintaining cancer stemness and progression of various diseases [194–196], further investigations are needed to explore how SCUBE2–HH signaling is involved in the progression of human pathologies, such as SHH-subgroup medulloblastoma [197]. Studies are also needed to develop treatments that can target SCUBE2-enhanced HH signaling.

Because enhancement of TGF-β signaling by SCUBE3 has been linked to a variety of cancers, understanding the molecular and structural basis of enhancement action could facilitate the development of more effective cancer therapy. In addition, a thorough molecular understanding of SCUBE3-mediated effects on BMP signaling could contribute to future treatments for craniofacial or bone defect in patients with SCUBE3 loss-of-function mutations. A number of critical DNA and protein reagents and many genetically modified animal models have already been established for investigating the SCUBE family. Further investigations of the molecular functions and structural characteristics of SCUBE proteins should include collaborations among basic and clinical researchers. Such collaborative projects may uncover the clinical potential and translational utility of secreted and membrane-bound SCUBE proteins as diagnostic, prognostic and/or therapeutic targets for human diseases.

## Data Availability

Not applicable.

## Abbreviations

ACS: Acute coronary syndrome
ADC: Antibody–drug conjugate
AIS: Acute ischemic stroke
AML: Acute myeloid leukemia
BMP: Bone morphogenetic protein
BMPR: BMP receptor
cbEGF: Calcium binding EGF
CR: Cysteine-rich
EC: Endothelial cell
EGCG: (–)-Epigallocatechin-3-gallate
EGF: Epidermal growth factor
EMT: Epithelial-mesenchymal transition
ENU: Ethyl-*N*-nitrosourea
FGF8: Fibroblast growth factor 8
FLT3: Fms-like receptor tyrosine kinase 3
HH: Hedgehog
HIF: Hypoxia-inducible factor
HOXA9: Homeobox A9
HUVECs: Human umbilical vein endothelial cells
IHH: Indian hedgehog
I/R: Ischemia–reperfusion
KO: Knockout
MAPK: Mitogen-activated protein kinase
MEIS1: Meis homeobox 1
MLL-r: Leukemia gene-rearranged
MMP: Matrix metalloproteinase
NSCLC: Non-small cell lung cancer
PAH: Pulmonary arterial hypertension
SCUBE: Signal peptide-complement C1r/C1s, Uegf, Bmp1 (CUB)-epithelial growth factor domain-containing protein
sCD40L: Soluble CD40 ligand
SHH: Sonic hedgehog
SNP: Single nucleotide polymorphism
STEMI: Stent thrombosis-elevation myocardial infarction
TGF-β: Transforming growth factor beta
VEGF: Vascular growth factor
VEGFR2: Vascular growth factor receptor 2
